# Optogenetic stimulation of the “Zusanli” acupoint alleviates inflammatory pain through active Wnt/β-Catenin and MAPK signaling pathway in rats

**DOI:** 10.1016/j.heliyon.2024.e39992

**Published:** 2024-10-30

**Authors:** Rong Chen, Meng Li, Mingxing Ding

**Affiliations:** a College of Veterinary Medicine, Huazhong Agricultural University, Wuhan, 430070, China; b College of Animal Science and Technology, Tarim University, Alar, Xinjiang, 843300, China

## Abstract

Inflammatory pain, an important form of common pain, negatively influences the quality of life. Pathway-selective optogenetic control is a popular tool in neuronal function research; however, attempts to modulate rodent behavior using pathway-selective optogenetics remain unverified. We developed a methodology for pathway-selective optogenetics in rats, focusing on the delivery of recombinant adeno-associated virus (rAAV) containing channelrhodopsin-2 (ChR2) injected at the “Zusanli” acupoints to the dorsal root ganglia (DRG) and toes, which is a part of the complex neuron network. Optogenetic stimulation of gamma-aminobutyric acid (GABA) projections to the “Zusanli” acupoints delivered several rAAV9-GAD1-ChR2-mcherry particles to the spinal cord horn (SCDH) and DRG and few rAAV9-GAD1-ChR2-mcherry particles to toes, thereby evoking an analgesic effect in complete Freund's adjuvant (CFA) rats similar to those of acupuncture. Furthermore, blue light stimulation (LED) and electroacupuncture (EA) reduced inflammatory pain in CFA rats, as evidenced by the changes in the paw edema, paw withdrawal latency (PWL), and paw withdrawal threshold (PWT) values. Further exploration of the mechanisms underlying this phenomenon revealed that optogenetic stimulation upregulates the decarboxylase-65 (GAD65) and decarboxylase-67 (GAD67) protein levels and downregulates the levels of GABA transporter-1 (GAT1) and GABA transporter-3 (GAT3) to alleviate inflammatory pain. The activation of the p38 mitogen-activated protein kinases (MAPK) and Wnt signal transduction pathways decreased the release of interleukin 6 (IL-6), interleukin 10 (IL-10), and tumor necrosis factor-α (TNFα). These findings indicate that optogenetic stimulation of the “Zusanli” acupoint alleviated inflammatory pain.

## 1 Introduction

Inflammatory and neuropathic pain, which affect more than 30 % of people worldwide, cause psychological distress and unhappiness, thereby exerting enormous economic burdens[1,2]. Neuropathic pain is a direct consequence of a lesion or disease affecting the somatosensory nervous system. In contrast, inflammatory pain arises from chemical/natural stimuli that damage tissue or inflammatory processes associated with surgery, osteoarthritis, and trauma that induce hyperalgesia, allodynia, and sympathetically maintained pain [3]. Chronic inflammatory pain is a common and challenging symptom to treat in clinical practice. Physical therapy and pharmacotherapy have remained the primary strategies for inflammatory pain management. However, these strategies are often ineffective. Moreover, they are associated with adverse effects and exhibit low efficacy, highlighting the importance of further research on pain [4]. Thus, pain has become an expanding topic of research in recent years.

Acupuncture, a basic technique in traditional Chinese medicine that involves the insertion of needles into specific acupoints, has been applied widely in medical treatment. Mast cells and nerve terminals have been observed in abundance at acupoints. These mast cells degranulate and release mediators that can sensitize the neurons to release the neurotransmitters or neuropeptides into the central nervous system when the needle is twirled, lifted, or connected to an electricity source[[5], [6], [7]].

Optogenetics, an approach that combines optics and genetics, was introduced recently for modulating cell activity through light stimulation at particular wavelengths. Optogenetics has exhibited excellent value in overcoming some of the limitations of other neuromodulation therapies [8]. Thus, it is a novel and readily available tool that facilitates the dissection of neural circuits into specific neuronal subpopulations within discrete nerve regions to elucidate their role in the pain pathway[[9], [10], [11]]. Channelrhodopsin-2 (ChR2), a light-sensitive protein commonly used in optogenetics, induces intracellular cation flow when activated by blue light. ChR2 was successfully transfected into dorsal root ganglia (DRG) and spinal cord neurons in a study by Zhang et al. resulting in cell activation [12]. The inhibitory neurotransmitter g-aminobutyric acid (GABA) regulates neuronal excitability[13,14]. GABA is synthesized from glutamate via the activity of glutamate decarboxylase (GAD). GAD has two isoforms: GAD65 and GAD67. GABA transporters (GATs) 1–4, the most abundant of which is GAT1, remove GABA from the postsynaptic cleft [15].

Previous studies have attempted to apply optogenetics to DRG and spinal cord neurons in rats treated with complete Freund's adjuvant (CFA). Light-emitting diode (LED) arrays were used in these studies to activate opsin ChR2, which is expressed transgenically or delivered through gene therapy.

Therefore, we constructed and identified rAAV9-GAD1-ChR2-mcherry; its expression in the T293 cells and rats was analyzed, and an inflammatory pain model was established by injecting CFA into rats and restoring inflammatory pain in rats through LED and electroacupuncture (EA) treatment. We investigated whether LED and EA could relieve inflammatory pain through the activation of the p38 MAPK and Wnt signal transduction pathways in the present study to determine whether these signal transduction pathways in the spinal cord play an important role in inducing inflammatory pain.

## 2 Materials and methods

### 2.1 Viral vector preparation and characterization

**Fig. 1:**
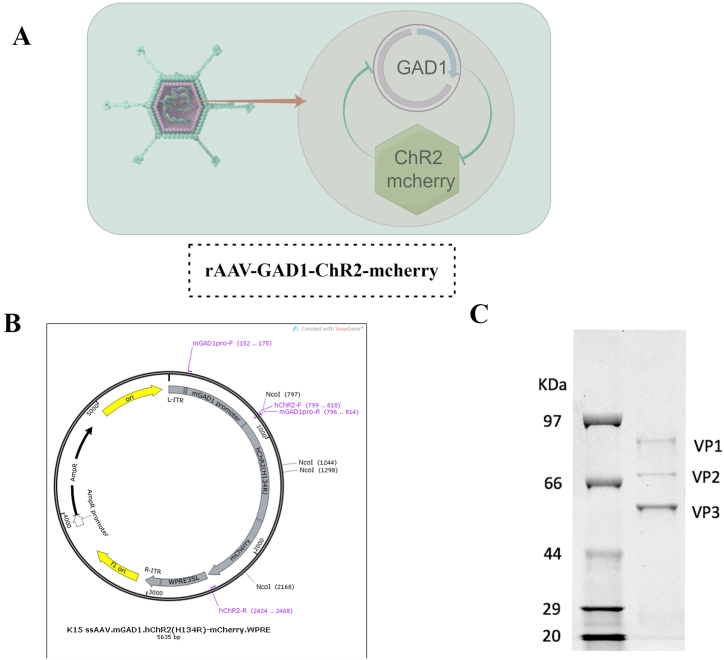
**Construction and detection of rAAV9-GAD1-ChR**_**2**_**-mcherry**. A: Flow Diagram. B: Plasmid pK15-GAD1-chR2-mcherry Construction. C: 10 % SDS-PAGE electrophoresis of the bands.

The adenoviral vector used in the present study was meticulously engineered to selectively target GABAergic neurons. The promoter for GAD1, which facilitated the expression of GAD1 in GABA neurons, was isolated from the genomic DNA of rats. Adeno-associated virus serotype 9 (AAV9) was used as the viral vector to construct the recombinant plasmids (rAAV9). Established molecular biology protocols were used to assemble the recombinant construct rAAV-GAD1-ChR2-mCherry (Fig. 1A). Kits (PG20161130) provided by Qiagen (CA, USA) were used to obtain endotoxin-free plasmid preparations of rAAV-GAD1-ChR2-mCherry.

### 2.2 Expression of rAAV9-GAD1-ChR_2_-mcherry in the T293 cells

The calcium phosphate transfection methodology was used to transfect the plasmids into T293 cells at a 1:1:1 M ratio for packaging. The transfected cells were collected 48 h post-transfection and lysed using sodium deoxycholate to achieve a final concentration of 0.5 %, in conjunction with multiple freeze/thaw cycles to ensure cell disruption. Low-speed centrifugation was performed to clarify the cell lysate. Affinity chromatography was performed using AVB Sepharose High-Performance columns (GE Healthcare, NJ) to purify the rAAV9 particles. The rAAV9 particles bound to the column were eluted with 50 mM glycine buffer and concentrated to achieve a final concentration of 300 ng/μL.

Sodium dodecyl sulfate-polyacrylamide gel electrophoresis (SDS-PAGE 10 %), followed by visualization using Coomassie Blue staining (Invitrogen), was performed to assess the purity of the rAAV preparations. Quantitative PCR (qPCR) with TaqMan technology (Life Technologies) was performed to quantify the viral titers of the rAAV preparations. The viral titers were determined accurately using a standard curve, ranging from 4.6 × 10^9^ to 1 × 10^13^ vector genomes (vg) [16].

### 2.3 Expression of ChR2-mcherry in rats

#### 2.3.1 Animals and animal grouping

SPF Sprague–Dawley rats (weighing 200 ± 20 g) were acquired from the Hubei Provincial Center for Animal Research, Chinese Academy of Sciences (SCXK [HU) 2020-0003]. The rats were group-housed while breeding with a maximum of six animals per cage (temperature: 25 ± 2 °C, humidity: 40–70 %). A reversed 12 h light/dark cycle was maintained, and food and water were provided *ad libitum*. All experimental protocols adhered to the Regulations on the Control of Laboratory Animals of the People's Republic of China.

The animals were randomly divided into eight groups (n = 6): the control (saline), CFA, CFA + EA, CFA + ChR_2_+LED (15 Hz) + Dicentrine (Dic), CFA + rAAV + LED (15 Hz), CFA + ChR_2_+LED (2 Hz), CFA + ChR_2_+LED (15 Hz), and CFA + ChR_2_+LED (60 Hz) group.

#### 2.3.2 Injection of rAAV-GAD1-ChR2-mcherry at the acupoints

Anesthesia was induced via the intraperitoneal injection of 1 % pentobarbital sodium. Fur was shaved off at the incision site of the bilateral “Zusanli” acupoints and sterilized with iodine and 75 % alcohol. Sterilized forceps and spring scissors were used to make a 0.1-mm incision at the level of the acupoint after taping the leg to the surgical table. A micro syringe (Hamilton) was used to insert a needle through the incision, and 1 μL (300 ng/μL) of the virus solution was injected into the “Zusanli” acupoints. Care was taken to minimize the damage to the skin and muscles.

### 2.4 Inflammatory pain model

The mechanisms underlying pain disorders have often been investigated using CFA-induced inflammatory pain [8]. The rats were anesthetized by administering 1 % of pentobarbital sodium through intraperitoneal injection. CFA (0.1 mL; Sigma-Aldrich, USA) was added subsequently through intraplantar injection into the right hind paw to establish the inflammatory pain model [17]. An equal volume of physiological saline was administered to the rats in the control group. The rats were returned to their cages for recovery immediately after injection.

### 2.5 LED and EA treatment

The LED and EA stimulation procedures were conducted as described in previous studies[18,19]. All rats in the LED group were subjected to LED stimulation. The intervention was commenced 30 min after the CFA injection (day 0), by attaching the LED light onto the bilateral Zusanli (ST36) acupoints. The parameters of the stimulator were as follows: 2, 15, and 60 Hz; 10 s on/off; and stimulation for 30 min, once a day.

The rats in the CFA + EA group were subjected to EA stimulation. The intervention was commenced 30 min after the CFA injection (day 0), and stainless steel needles (0.18 mm × 13 mm) were inserted into the bilateral Zusanli (ST36) acupoints. The needles were connected to a WQ-6F Acupuncture Point Nerve Stimulator (Xin Donghua Co. Ltd., Beijing, China) with the following parameters: 15 Hz stimulation for 30 min once a day.

#### 2.5.1 Paw withdrawal threshold (PWT)

The PWT was determined as described in a previous study [20]. The rats were acclimatized for a minimum of 20 min on the test day. The von Frey test was conducted to evaluate mechanical allodynia after LED stimulation. The withdrawal response includes rapid flinching, withdrawal of the paw, or spreading of the toes. The Von Frey test was conducted by a single examiner blinded to whether the mice expressed opsin. A series of calibrated Von Frey filaments (Stoelting, Wood Dale, IL, USA) were applied perpendicular to the plantar surface of the left hind paw with sufficient force such that the filament was bent. The test was repeated thrice in each rat at 5-min intervals; the mean value was calculated subsequently.

#### 2.5.2 Paw withdrawal latency (PWL)

PWL was assessed as described in a previous study [21]. The rats were acclimatized for a minimum of 20 min on the test day. The radiant heat source of the test instrument (37,370, UGO, Italy) was positioned on the ventral surface of the hind paw, and the elapsed time between the application of heat and the withdrawal response was recorded as the PWL. PWL was recorded thrice at each time point at 2-min intervals. The mean value was calculated and used for further calculations. The radiant heat and cut-off time were set at 35 °C and 20 s, respectively.

#### 2.5.3 Paw edema

A plethysmometer (Model 7150, Italy) was used to evaluate paw edema at 0, 1, 3, 5, 7, 25, and 73 h after CFA injection. This test was performed as described in a previous study [22]. The right hind paw of each rat was immersed in a container containing the electrolyte solution till the boundary between the hairy and non-hairy skin. The volume displacement was determined automatically. The paw volume obtained from two consecutive trials was averaged. Data are expressed as the volume displaced in milliliters (mL). The analyses were performed using the difference in times with the first baseline assessment as the reference.

#### 2.5.4 Reverse transcription quantitative PCR (RT-qPCR)

qPCR was performed to quantify the gene transcripts of *GAD65*, *GAD67*, *GAT1*, and *GAT3* in the CFA rats sacrificed 7 days after treatment with LED and EA. TRIzol reagent (Invitrogen, Carlsbad, CA USA) was used following the manufacturer's instructions to extract total RNA. Complementary DNA (cDNA) was synthesized using Prime Script Reverse Transcriptase (Takara, Japan). RT-qPCR was performed using SYBR Premix Ex Taq™ II (Takara, Japan) to amplify the cDNA using the following primer sequences. *GAT1*: Forward primer, ACCGCTGCTTCTCCAACTAC; Reverse primer**,** GCTGCCCCAAAGTCTGATGT; *GAT3*: Forward primer, GGAGGGAGGTCCTCATCCTT; Reverse primer**,** CGCCCCTTGAGAGTTCTGTT; *GAD65*: Forward primer, TCGCGTTCACATCAGAGCAT; Reverse primer**,** GGCAAACAGTACCTCTCGTCA; *GAD67*: Forward primer, AAGCACCAAATAGGGGAGCC; Reverse primer**,** TCAGCCATTCGCCAGCTAAA. The amount of mRNA in each sample was normalized to that of GAPDH. The 2−DDCt method was used to calculate the relative amount of target gene transcripts as described in a previous study [21]. The values were used to calculate the mean and standard error of the relative expression of the target mRNA in the DRG and SCDH of the CFA-treated mice.

#### 2.5.5 Enzyme-linked immunosorbent assay (ELISA)

An ELISA kit (MM-0163M1, MM-0132M1, MEIMIAN, China) was used to measure the serum and DRG interleukin 6 (IL-6), tumor necrosis factor-α (TNF-α), and IL-β. Serum was separated from the blood sample by centrifuging at 2000 rpm for 5 min. The tissue (0.5 g) was weighed and homogenized in 4.5 mL of phosphate-buffered saline (PBS) using a tissue homogenizer. The supernatant was collected by centrifuging at 3000 rpm for 10 min. ELISA was performed following the manufacturer's protocol. The samples were added to each well and incubated at 37 °C for 30 min. The colorant was added subsequently and incubated at 37 °C for 10 min. Subsequently, the termination solution was added immediately. The optical density values were measured using an enzyme marker at 450 nm. The serum and DRG IL-6, TNF-α, and IL-β levels were calculated through the gradient standard.

### 2.6 Western blotting

The mice were sacrificed after the behavior tests on day 14. The rats were anesthetized with 10 % choral hydrate at 0.35 mL/100 g body weight and the L4–6 segments of the DRG were removed and stored at −80 °C. The tissue samples were homogenized in radioimmunoprecipitation assay buffer (Solarbio, Beijing, China) and placed on ice for 30 min for lysis. The solution was centrifuged at 12,000 *g* for 20 min at 4 °C, and the supernatant was transferred to a lysis buffer. The Enhanced BCA Protein Assay Kit (Beyotime, Jiangsu, China) was used in accordance with the manufacturer's instructions to determine the total protein levels. Protein samples (30 μg) were separated using SDS-PAGE (10 %) and were electrophoretically transferred to polyvinyl difluoride membranes (Bio-rad, USA). The membrane was rinsed and incubated with primary (anti-GAD65, 1:1000, A0971, ABclonal; anti-GAD67, 1:1000, A1457, ABclonal; anti-GAT1, 1:1000, A23077, ABclonal) and secondary antibodies. The membrane was rinsed with 0.1 % Tween-20 to remove excess secondary antibodies. The Omni-ECL™ Femto Light Chemiluminescence Kit was used following the manufacturer's instructions to detect the secondary antibodies. The density of reactions was measured using Image J software.

### 2.7 Immunofluorescence

Normal saline and 4 % paraformaldehyde in 0.1 PBS (Solarbio, China) were infused transcardially for fixation. Tissues containing nerve terminals, DRG, spinal cord, and toes were carefully removed. The optimal cutting temperature compound (OCT)-embedded blocks were sectioned at a thickness of 20 μm. The sections were rinsed with tris-buffered saline-tween-20 - saline (TBST) at least three times and blocked with 10 % normal goat serum for 1 h at 37 °C and incubated overnight at 4 °C with the following antibodies: anti-GAD65 (1:1000, A0971, ABclonal), anti-GAD67 (1:1000, A1457, ABclonal), anti-GAT1 (1:1000, A23077, ABclonal), and Rabbit anti-β-actin (1:100, #N4142, Sigma-Aldrich). The secondary antibodies used was horseradish peroxidase (HRP)-labeled goat anti-rabbit IgG (1:500, AC047, ABclonal) at 37 °C for 1 h. Leica TCS SP5 confocal scanning laser microscope was used for imaging. ImageJ software was used for the quantitative analyses of positive cells.

### 2.8 Statistical analysis

All data are presented as the mean ± standard error of the mean (x± SEM). All statistical analyses were performed using SPSS 20.0 software. The between-group variance was evaluated through one-way analysis of variance (ANOVA), followed by Tukey's post-test. A *P-*value <0.05 was considered statistically significant.

## 3 Results

### 3.1 Construction and identification of rAAV9-GAD1-ChR_2_-mcherry

Fig. 1A illustrates the construction of the rAAV9-GAD1-ChR2-mcherry vector. Fig. 1B illustrates the plasmid. Fig. 1C illustrates the identification of rAAV9-GAD1-ChR2-mcherry. The titer and purity of AAV9-GAD1-chR2-mcherry were 3.62E+13 GC/mL and 97.2 %, respectively.

### 3.2 Expression of rAAV9-GAD1-ChR_2_-mcherry in the T293 cells

**Fig. 2:**
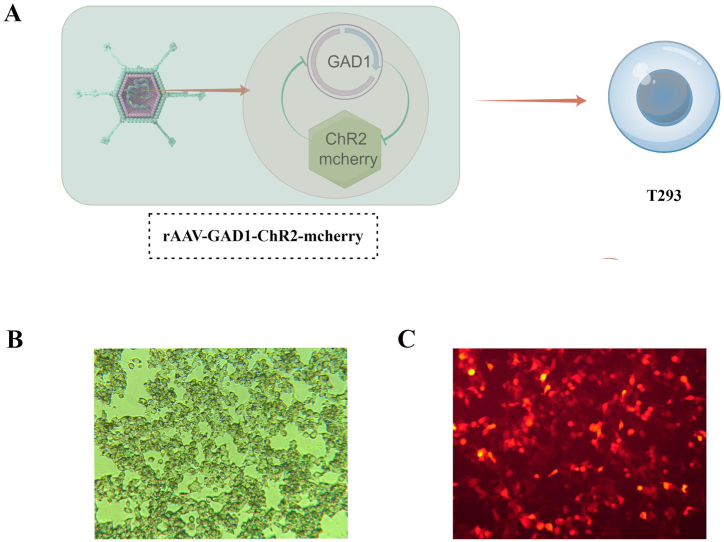
The expression of AAV9-GAD1-ChR2-mcherry in T293 cell. A: Flow Diagram. B: Optical microscope image of the expression of AAV9-GAD1-ChR2-mcherry in T293 cell (40 × ). C: Fluorescence microscope picture of T293 cell (40 × ).

Fluorescence microscopy was performed to detect the expression of rAAV9-GAD1-ChR2-mcherry in the 293T cells (Fig. 2A). The results illustrated in Fig. 2B and C confirmed the expression of ChR2 protein, as evidenced by red fluorescence, in the 293T cells.

### 3.3 Expression of rAAV9-GAD1-ChR_2_-mcherry in rats

**Fig. 3:**
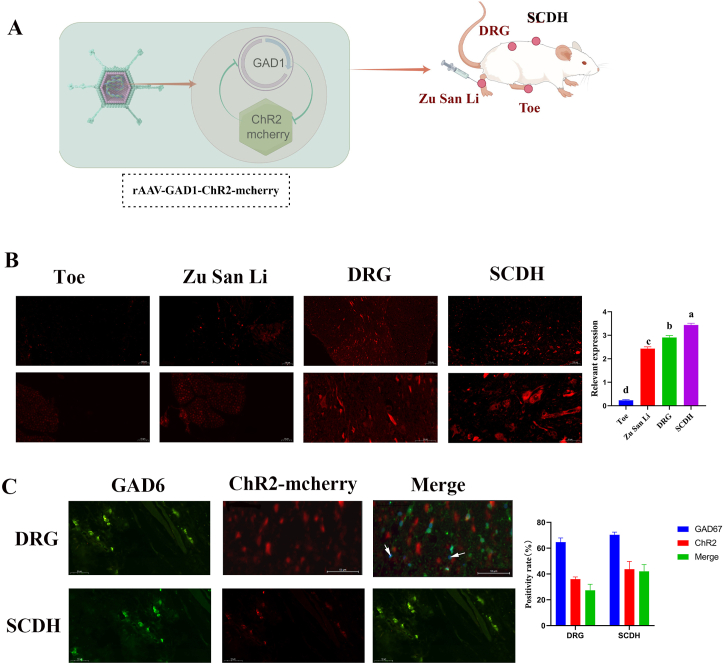
The expression of rAAV9-GAD1-ChR2-mcherry in rats. A: Flow Diagram. B: Fluorescence microscope picture of expression of rAAV9-GAD1-ChR2-mcherry in rats, First-row bar is 100 μm, Second-row bar is 50 μm. C: Colocalization of GAD67 and ChR2-mcherry in DRG and SCDH. The white arrow was Colocalization.

The expression of rAAV9-GAD1-ChR2-mcherry in the toe, Zusanli, DRG, and SCDH sites was further analyzed. Fig. 3A represents the experiment using a flow diagram. The rAAV-GAD1-ChR2-mCherry (Fig. 3A) were injected into the bilateral “Zusanli” acupoints on day 1. The tissues were harvested from the sacrificed animals 15 days post-injection to assess the distribution and expression of ChR2 in the toe, Zusanli, DRG, and SCDH. Staining for mCherry fluorescence was performed subsequently. Fluorescence microscopy revealed a gradual increase in red fluorescence across the toe, Zusanli, DRG, and SCDH (*P* < 0.05). Notably, the intensity of red fluorescence in the SCDH was significantly higher than that in the toe, Zusanli, and DRG (Fig. 3B). The colocalization of GAD67 and ChR2-mcherry in DRG and SCDH was further analyzed using a fluorescence microscope; GAD67 and ChR2 were labeled with green and red dyes, respectively. GAD67 and ChR2 were expressed in the DRG and SCDH of rats (Fig. 3C).

### 3.4 Effects of different frequencies of LED and different stimulation times on PWL in CFA rats

**Fig. 4:**
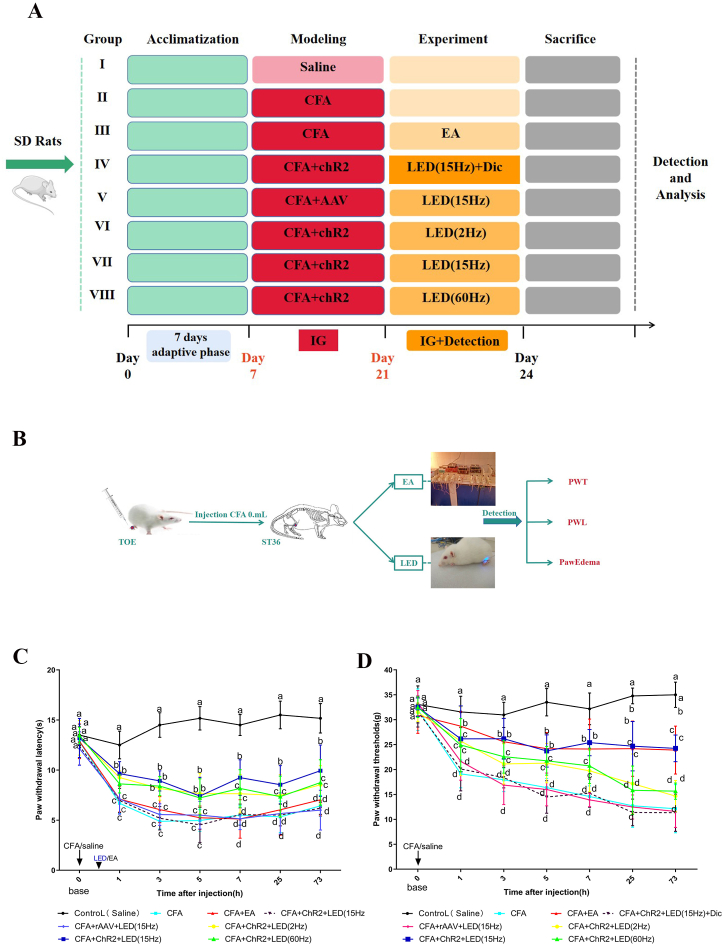
**Behavioral (PWL and PWT) of rats in each group at different time points.** A**.** Experimental design B**.** Experimental flow chart; C**.** PWL value of rats in each group at different time-points; D. PWT value of rats in each group at different time-points; Different letters were significantly different in each group at different time points (*p* < 0.05).

Fig. 4A and B presents the results of the PWL test of CFA rats treated with LED or EA and the experimental design and flow chart.

The PWL of the rats was measured at 0, 1, 3, 5, 7, 25, and 73 h. In terms of PWL, no significant difference were observed between the groups before infection with CFA (*P*﹥0.05; Fig. 4C). A significant decrease in PWL in the other groups compared with the control group, which received saline, was observed following CFA injection (1, 3, 5, 7, 25, and 73 h). In addition, the PWL of the CFA, CFA + rAAV + LED (15 Hz), and CFA + ChR2+ LED (15 Hz) + Dic groups was significantly lower than those of the CFA + EA, CFA + ChR2+LED (2 Hz), CFA + ChR2+LED (15 Hz), and CFA + ChR2+LED (60 Hz) groups (*P* < 0.01). Thus, the LED and EA intervention increased the pain thresholds in rats. The PWL of the CFA + EA and CFA + ChR2+LED (15 Hz) groups were restored similarly; thus, no significant differences were observed between the CFA + EA and CFA + ChR2+LED (15 Hz) groups in terms of pain reduction(*P*﹥0.05). However, the PWL in the CFA + ChR2+LED (2 Hz) and CFA + ChR2+LED (60 Hz) groups were similar and lower than those in the CFA + EA and CFA + ChR2+LED (15 Hz) groups.

### 3.5 Effects of different frequencies of LED and different stimulation times on PWT in CFA rats

PWT of the rats in each group was measured at 0, 1, 3, 5, 7, 25, and 73 h. PWT decreased significantly at 73 h after the CFA injection (*P* < 0.05; Fig. 4D). Similar to the effect of LED on PWL, the PWT in the CFA + EA and CFA + ChR2+LED (15 Hz) groups also increased. However, different frequencies (2, 15, and 60 Hz) and different LED stimulation times exhibited varying effects on PWT. The PWT of the CFA, CFA + rAAV + LED (15 Hz), and CFA + ChR2+ LED (15 Hz) + Dic groups were significantly lower than those of the CFA + EA, CFA + ChR2+LED (2 Hz), CFA + ChR2+LED (15 Hz), and CFA + ChR2+LED (60 Hz) groups (*P* < 0.05). The PWT of the CFA + EA and CFA + ChR2+LED (15 Hz) groups were restored similarly. However, the PWT in the CFA + ChR2+LED (2 Hz) and CFA + ChR2+LED (60 Hz) groups were similar and lower than those to those in the CFA + EA and CFA + ChR2+LED (15 Hz) groups.

### 3.6 Effects of different frequencies of LED and different stimulation times on paw edema in CFA rats

Paw edema values were measured 0, 1, 3, 5, 7, 25, and 73 h post-treatment across all groups. A significant time-dependent increase in paw edema was observed in the CFA group compared with the control group (Fig. S1; *P* < 0.05), confirming the successful establishment of the CFA model. A significant increase in the paw edema compared with that in the control group, which received saline injections, was observed in all treatment groups following CFA injection (at 1, 3, 5, 7, 25, and 73 h). The paw edema values observed in the CFA, CFA + rAAV + LED (15 Hz), and CFA + ChR2+ LED (15 Hz) + Dic groups were significantly higher than those observed in the CFA + EA, CFA + ChR2+LED (2 Hz), CFA + ChR2+LED (15 Hz), and CFA + ChR2+LED (60 Hz) groups (*P* < 0.05). Thus, LED and EA decreased paw edema in rats. The paw edema values were 2.098 and 2.138 in the CFA + EA and CFA + ChR2+LED (15 Hz) groups, respectively, indicating no significant differences. However, the paw edema values in the CFA + ChR2+LED (2 Hz) and CFA + ChR2+LED (60 Hz) groups were similar and higher than those in the CFA + EA and CFA + ChR2+LED (15 Hz) groups (*P* < 0.05; Fig. 4C).

Thus, PWL, PWT and paw edema exhibited similar tendencies in CFA rats subjected to LED and EA treatment.

### 3.7 GAT1 expression in the DRG and SCDH of CFA rats treated with LED

The relative quantity of *GAT1* expression in the DRG and SCDH was evaluated on day 14 to determine the mRNA and protein levels and colocalization using RT-qPCR, western blotting, and immunofluorescence.

**Fig. 5:**
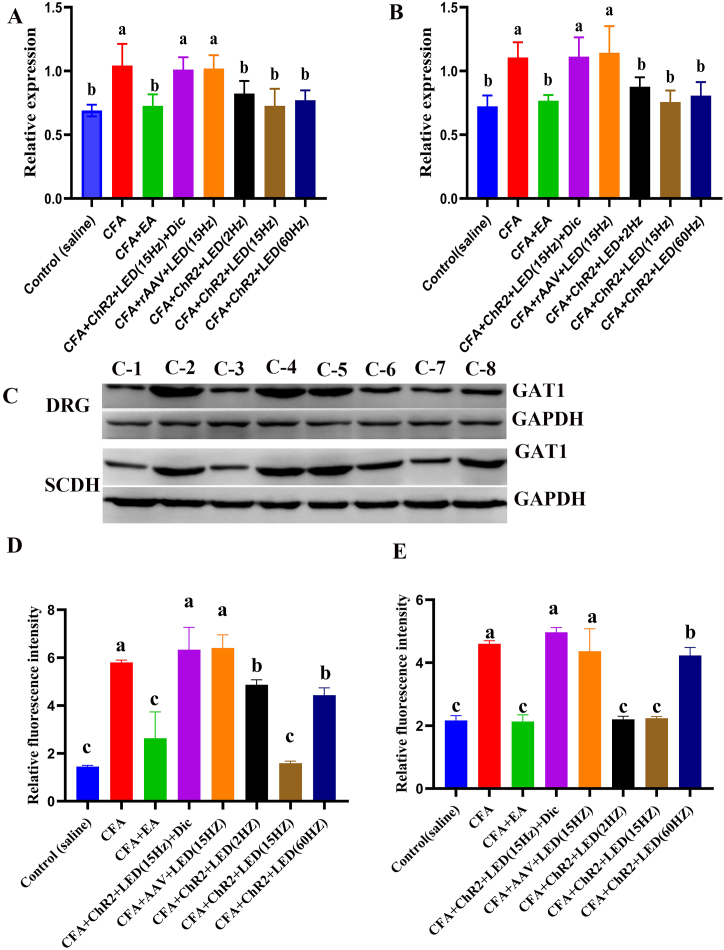
**The effect of LED and EA on rats' GAT1 expression in DRG and SCDH.** A. qRT-PCR results of *GAT1* expression in the DRG of rats; B. qRT-PCR results of *GAT1* expression in the SCDH of rats; Different letters were significantly different in each group (*P* < 0.05). C. Western blotting images of GAT1 in rats from different groups; C-1:Control(saline), C-2:CFA, C-3:CFA + EA, C-4:CFA + ChR_2_+LED (15 Hz) + Dic, C-5:CFA + rAAV + LED (15 Hz), C-6:CFA + ChR_2_+LED (2 Hz), C-7:CFA + ChR_2_+LED (15 Hz), and C-8:CFA + ChR_2_+LED (60 Hz); D. Relative fluorescence intensity of GAT1 expression in DRG and SCDH of rats; Different letters were significantly different in each group (*P* < 0.05); E. Relative fluorescence intensity of GAT1 expression in DRG and SCDH of rats; Different letters were significantly different in each group (*P* < 0.05).

The relative expression of *GAT1* in the CFA group was higher than that in the control group (*P* < 0.01). However, LED and EA stimulation downregulated the expression of *GAT1* compared with that in the CFA, CFA + rAAV + LED (15 Hz), or CFA + ChR2+LED (15 Hz) + Dic groups (*P* < 0.01). No marked changes in the relative expression of *GAT1* in CFA rats stimulated with LED and EA were observed, indicating that pain was restored following LED and EA stimulation (Fig. 5A–B).

GAT1 protein expression in the DRG and SCDH was also detected on day 14 after CFA or saline injection, and after LED and EA stimulation. GAT1 protein was expressed at low levels in the control group. However, high expression of GAT1 protein was observed in the CFA group. GAT1 protein expression in the DRG and SCDH was downregulated after LED and EA stimulation (Fig. 5C, Fig. S2).

Immunofluorescence revealed similar expression of *GAT1* and GAT1protein expression in the DRG and SCDH of rats. Compared with that in the CFA group, the fluorescence intensity in DRG was reduced by 1.20, 3.64, 1.3, and 2.23 times in the CFA + ChR2+LED (2 Hz), CFA + ChR2+LED (15 Hz), CFA + ChR2+LED (60 Hz), and CFA + EA groups, respectively. The fluorescence intensity in the SCDH reduced by 2.09, 2.06, 1.09, and 2.15 times in the CFA + ChR2+LED (2 Hz), CFA + ChR2+LED (15 Hz), CFA + ChR2+LED (60 Hz), and CFA + EA groups, respectively (Fig. 5D–E, Fig. S3).

Thus, GAT1 expression in the DRG and SCDH of CFA rats decreased after LED and EA treatment. The findings of the CFA + ChR2+LED (15 Hz) and CFA + EA groups were similar to those of the CFA group.

### 3.8 GAT3 expression in the DRG and SCDH of CFA rats treated with LED and EA

GAT3 expression in the DRG and SCDH was also tested using RT-qPCR, western blotting, and fluorescence.

The relative expression of *GAT3* in the CFA group was higher than that in the control group (*P* < 0.01). However, LED and EA stimulation downregulated the expression of *GAT3* compared with that in the CFA group (*P* < 0.05). No marked changes in the relative expression of *GAT3* were observed in CFA rats stimulated with LED or EA (Figs. S4A–B).

GAT3 protein expression in the DRG and SCDH was also evaluated 14 days after CFA or saline injection, as well as after LED and EA stimulation. GAT3 was expressed at low levels in the control group. However, high expression of GAT3 protein was observed in the CFA, CFA + rAAV + LED (15 Hz), and CFA + ChR2+LED + Dic groups. LED and EA stimulation downregulated overexpression of the GAT3 protein in the DRG and SCDH (Figs. S5A–C).

Immunofluorescence revealed similar relative expression of *GAT3* and GAT3 proteins in the DRG and SCDH. Compared with that in the CFA group, the fluorescence intensity in the DRG was reduced by 1.20, 3.64, 1.3, and 2.23 times in the CFA + ChR2+LED (2 Hz), CFA + ChR2+LED (15 Hz), CFA + ChR2+LED (60 Hz), and CFA + EA groups, respectively. The fluorescence intensity in the SCDH was reduced by 2.01, 3.17, 1.22, and 3.28 times in the CFA + ChR2+LED (2 Hz), CFA + ChR2+LED (15 Hz), CFA + ChR2+LED (60 Hz), and CFA + EA groups, respectively (Figs. S6A–B).

Thus, GAT3 expression in the DRG and SCDH of CFA rats decreased after LED and EA treatment.

### 3.9 GAD65 expression in the DRG and SCDH of CFA rats treated with LED and EA

The relative expression of GAD65 in the DRG and SCDH was evaluated on day 14 after CFA injection.

**Fig. 6:**
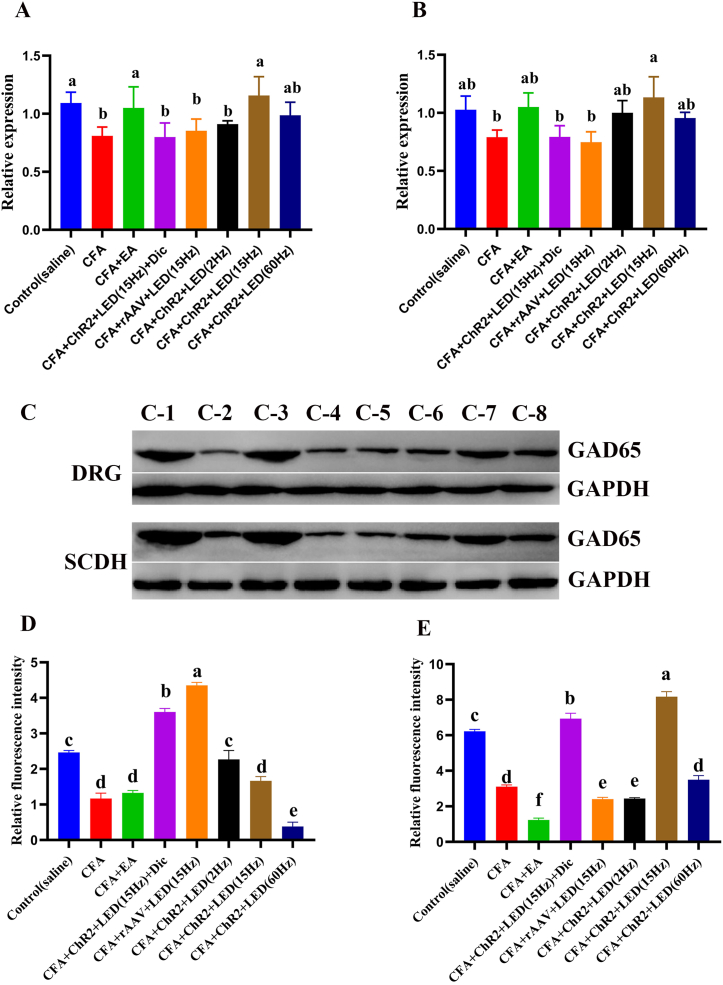
**The effect of LED and EA on rats' GAD65 expression in DRG and SCDH.** A. qRT-PCR results of *GAD65* expression in the DRG of rats; B. qRT-PCR results of *GAD65* expression in the SCDH of rats; Different letters were significantly different in each group (*P* < 0.05). C. Western blotting images of GAD65 in rats from different groups; C-1:Control(saline), C-2:CFA, C-3:CFA + EA, C-4:CFA + ChR_2_+LED (15 Hz) + Dicentrine (Dic), C-5:CFA + rAAV + LED (15 Hz), C-6:CFA + ChR_2_+LED (2 Hz), C-7:CFA + ChR_2_+LED (15 Hz), and C-8:CFA + ChR_2_+LED (60 Hz); D. Relative fluorescence intensity of GAD65 expression in DRG and SCDH of rats; Different letters were significantly different in each group (*P* < 0.05); E. Relative fluorescence intensity of GAD65 expression in DRG and SCDH of rats; Different letters were significantly different in each group (*P* < 0.05).

RT-qPCR revealed that the relative expression of *GAD65* in DRG was downregulated in the CFA group compared with the control group (*P* < 0.05). However, LED and EA stimulation upregulated the *GAD65* gene expression compared with that in the CFA group (*P* < 0.05). Interestingly, the expression of *GAD65* in the DRG of the CFA + ChR2+LED (15 Hz) and CFA + EA groups was similar. The relative expression of *GAD65* in the DRG was similar to that in the SCDH (Fig. 6A and B).

Western blotting revealed the GAD65 protein expression in the DRG and SCDH of rats. High expression of GAD65 was observed in the control group. However, the expression level of GAD65 protein was lower in the CFA group. The GAD65 protein expression in the DRG and SCDH of rats was upregulated by LED and EA stimulation (Fig. 6C, Fig. S7).

Immunofluorescence revealed similar relative expression of *GAD65* and GAD65 proteins in the DRG and SCDH of rats. Compared with that in the CFA group, the fluorescence intensity in the DRG was increased by 1.44, 2.03, 1.73, and 1.86 times in the CFA + ChR2+LED (2 Hz), CFA + ChR2+LED (15 Hz), CFA + ChR2+LED (60 Hz), and CFA + EA groups, respectively (Fig. 6D and E; Fig. S8).

In summary, GAD65 expression in the DRG and SCDH of CFA rats increased after LED and EA treatment.

### 3.10 GAD67 expression in the DRG and SCDH of CFA rats treated with LED and EA

GAD67 expression in the DRG and SCDH of rats was tested using RT-qPCR, western blotting, and fluorescence.

The relative expression of *GAD67* in the CFA group was lower than that in the control group (*P* < 0.01). However, LED and EA stimulation upregulated *GAD67* expression, compared with that in the CFA group (*P* < 0.01). No marked changes in the relative expression of *GAD67* was observed in CFA rats stimulated with LED or EA, indicating that the relative expression of *GAD67* in the LED-and EA stimulation groups was similar (Fig. S9).

Western blotting was performed to measure GAD67 protein expression in the DRG and SCDH of CFA rats. GAD67 protein was expressed at a low level in the CFA group. However, its expression was high in the CFA, CFA + rAAV + LED (15 Hz), and CFA + ChR2+LED + Dic groups. The expression of the GAD67 protein in the DRG and SCDH of rats was upregulated by LED and EA stimulation (Fig. S10).

Immunofluorescence revealed similar relative expression of *GAD67* and GAD67 proteins in the DRG and SCDH of rats. Compared with that in the CFA group, the fluorescence intensity in the DRG was increased by 1.17, 1.39, 1.32, and 1.43 times in the CFA + ChR2+LED (2 Hz), CFA + ChR2+LED (15 Hz), CFA + ChR2+LED (60 Hz), and CFA + EA groups, respectively (Fig. S11).

Thus, GAD67 expression in the DRG and SCDH of CFA rats increased after LED and EA treatment.

### 3.11 Changes in the cytokine levels in the DRG and serum of CFA rats after treatment with LED and EA

Cytokines such as TNF-α, IL-6, and IL-1β are key mediators of inflammation. The serum and DRG concentrations of TNF-α, IL-6, and IL-1β were quantified using ELISA kits. Significant elevations in the levels of IL-6, IL-10, and TNF-α were observed in the CFA group compared with the control group (*P* < 0.05). Moreover, a notable decrease in the concentrations of IL-6, IL-10, and TNF-α was observed in the DRG and serum of rats compared with that in the CFA group (*P* < 0.05). However, no significant differences were observed between the LED and EA treatments (*P* > 0.05) or LED treatments at frequencies of 2, 15, and 60 Hz (*P* > 0.05) (Fig. S12).

### 3.12 Effect of LED treatment on the MAPK and Wnt/β-Catenin signaling pathway in the SCDH of CFA rats

**Fig. 7:**
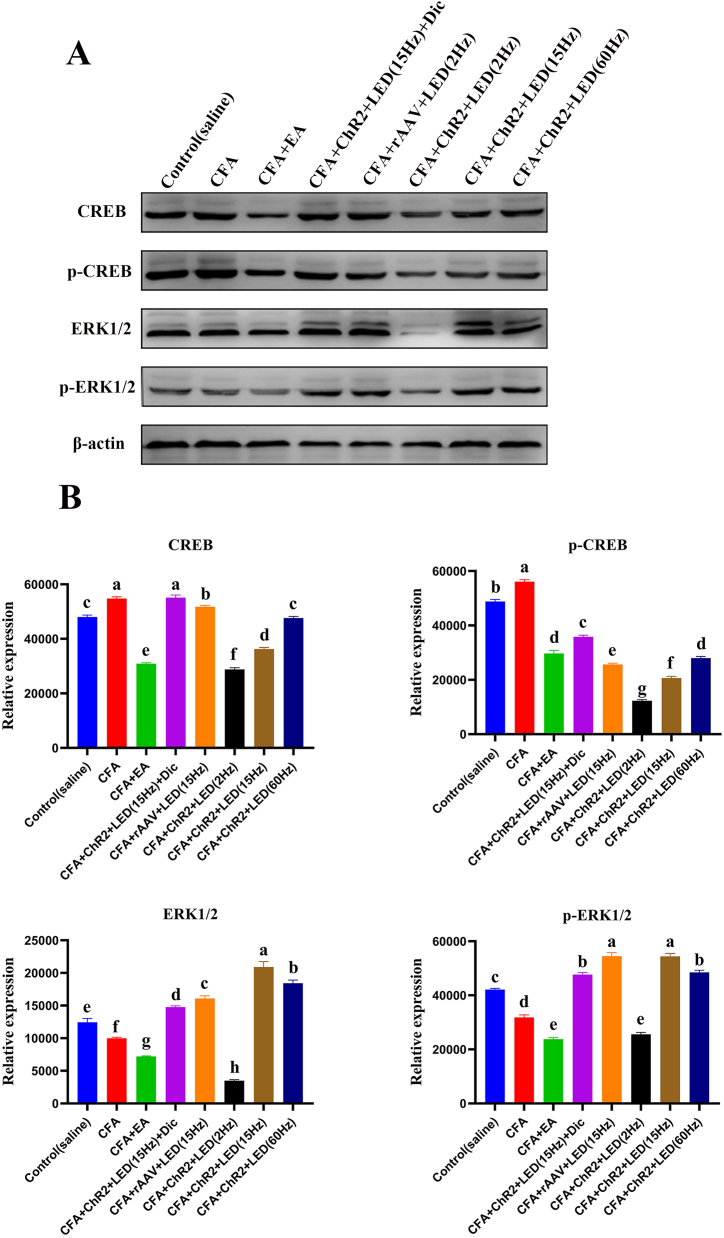
**Effect of LED and EA treatment on MAPK Signaling pathway in the SCDH of CFA-injected rats.** A. Western blot analysis revealed that LED and EA reduce levels of ERK1/2, pERK1/2, CREB and pCREB protein in the SCDH 72 h after CFA injection. B. Quantification of CREB, pCREB, ERK1/2 and pERK1/2 normalized against β-actin. Each bar represents mean ± SEM (n = 3). Different letters were significantly different in each group (P < 0.05).

All rats were anesthetized after treatment with LED and EA, and SCDH (L4–6) samples were collected to determine the effect of LED and EA on ERK1/2, pERK1/2, CREB, and pCREB proteins. Western blotting revealed that compared with those in the control group, the CREB and pCREB protein levels were upregulated 72 h after CFA injection (*P* < 0.05). LED and EA treatment greatly inhibited the CFA-induced increase in CREB and pCREB protein expression (*P* < 0.05); however, no substantial effect was observed in the CFA + rAAV + LED (15 Hz) and CFA + ChR2+LED(15Hz) + Dic groups (Fig. 7A and B). Western blotting also revealed that compared with those in the control group, the ERK1/2 and pERK1/2 protein levels were downregulated 72 h after CFA injection (*P* < 0.01). LED and EA treatment restored the CFA-induced increase in ERK1/2 and pERK1/2 protein expression to a significant extent (*P* < 0.01) (Fig. 7A and B).

The protein expression of key genes in the Wnt/β-catenin signaling pathway was also analyzed (Fig. S13). displays the expression of key proteins in the Wnt/β-Catenin signaling pathway following the administration of various agents, with β-actin protein expression remaining consistent across all groups (*P* > 0.05), serving as the internal reference. Western blotting also revealed an upregulation of Wnt and β-catenin protein levels. In contrast, the GSK3β protein levels were downregulated 72 h post-CFA injection compared with that in the control group (*P* < 0.05). Treatment with LED and EA significantly inhibited the CFA-induced increase in GSK3β protein expression (*P* < 0.05) and decreased Wnt and β-catenin protein levels compared with that in the CFA group (*P* < 0.05). However, the GSK3β protein expression in the CFA group was similar to that in the CFA + rAAV + LED (2 Hz) and CFA + ChR2+LED + Dic groups. Thus, treatment with LED and EA significantly ameliorated the CFA-induced changes in Wnt and β-catenin protein expression in the SCDH (L4–6) (*P* < 0.01).

## 4 Discussion

Owing to its prevalence and variety of associated diseases, pain represents a significant challenge in clinical settings. Thus, developing effective pain management strategies is crucial. Traditional pain management involves pharmacological treatments, behavioral rehabilitation, and neurosurgical interventions (such as neurotomy and rhizotomy) [2]. Optogenetic manipulation is an innovative approach for the management of pain that has exhibited substantial potential for addressing the limitations of conventional neuromodulation therapies. Notably, its application also extends to anxiety research [8]. The present study demonstrated that the injection of rAAV9-GAD1-ChR2-mCherry into the right hind paw induced progressive expression of ChR2 at the toe, Zusanli acupoints, DRG, and SCDH.

AAV vectors are favored for optogenetic manipulation owing to their ability to facilitate gene expression across extensive regions; thus, AAVs are a valuable tool for ChR2 expression, with demonstrated efficacy and safety for therapeutic gene transfer in humans[23,24]. AAVs were used to express ChR2 with red mCherry protein and the GAD promoter in 293T cells and rats in the present study. GAD67, a key enzyme in GABA synthesis, reflects the functional state of the GABA transmitter system. The co-localization of GAD67 and ChR2-mcherry was observed in the DRG and SCDH in the present study. These findings indicate that AAVs injected into the Zusanli activate GABA neurons in the DRG and SCDH of the rats.

Different frequencies of LED (2, 15, and 50 Hz) exhibited varying analgesic effects on pain parameters such as PWT, PWL, and paw edema in inflammatory pain models. The analgesic efficacy of LED is correlated with its frequency, with the effects of 15 Hz being superior to those of 2 and 60 Hz, similar to the effects of EA, a traditional Chinese analgesic therapy. EA is effective for various inflammatory conditions with minimal side effects[22,25]. Furthermore, it can effectively reduce mechanical and thermal hyperalgesia associated with peripheral inflammation[19,20]. Stimulation frequency plays a critical role in optogenetic manipulation as it affects the release of neurotransmitters and neuropeptides. Low-frequency optogenetic stimulation triggers the release of amino acid neurotransmitters, whereas high-frequency stimulation triggers the release of neurotransmitters and neuropeptides [26]. Dopaminergic neurons in the ventral tegmental area exhibit different release patterns following low-frequency (5 Hz) and high-frequency (50 Hz) optogenetic stimulation [27]. The present study revealed that the analgesic effect of LED at 15 Hz was better than that of other frequencies.

The present study also investigated whether LED with different stimulation frequencies exerted different analgesic effects on inflammatory pain and whether LED with varying stimulation frequencies mediated the GAT1, GAT3, GAD65, and GAD67 levels in the SCDH and DRG. GAT1 and GAT3 are observed exclusively in the brain. The expression of GAT1, which is widely distributed throughout the brain, closely follows that of the GABAergic pathways [28]. GAT3 is predominantly expressed in the distal astrocytic processes in direct contact with GABAergic neurons, with more restricted localization. Thus, GAT1 is primarily located in presynaptic regions. GAT3 is located mainly in the distal astrocytic processes close to the synapse[15,29]. GAT1 and GAT3 expression in the DRG and SCDH of CFA rats decreased following treatment with LED and EA. The CFA + ChR2+LED (15 Hz) and CFA + EA groups exhibited results similar to those of the CFA group.

GAD65 and GAD67 are two isoforms of GAD. GAD65 is preferentially located in nerve terminals, whereas GAD67 is ubiquitously expressed in GABAergic neurons. GAD65 specializes in the synthesis of GABA under short-term conditions. Presynaptic GABA receptors metabotropically mediate a negative feedback mechanism on GABA release. In contrast, postsynaptic GABA receptors can serve as metabotropic (GABAB) and ionotropic (GABAA) receptors, and usually mediate hyperpolarization of the cell [30]. The present study revealed that GAD65 and GAD67 expression in the DRG and SCDH of CFA rats reduced after treatment with LED and EA. The CFA + ChR2+LED (15 Hz) and CFA + EA groups exhibited results similar to those of the CFA group.

p38 MAPK activation is linked to allodynia/hyperalgesia induced by inflammation. The c-Jun N-terminal kinase (JNK) pathway plays a crucial role in developing and maintaining chronic pain [31]. JNKs, including JNK1, JNK2, and JNK3, are essential members of the MAPK family. Activating JNK1 and JNK2 is important in developing and maintaining chronic pain. JNK1/2 is activated in the spinal dorsal horn following the intraplantar injection of CFA [32]. In addition to the decreased GABAB receptor expression in SCDH under CFA-inflammatory conditions, the ERK1/2 and pERK1/2 protein levels were also decreased under CFA-inflammatory conditions in the present study. LED and EA treatment significantly restored the CFA-induced increase in ERK1/2 and pERK1/2 protein expression. LED-induced GABAB receptor activation further facilitated the activation of ERK by enhancing ERK phosphorylation in CFA-stimulated SCDH. GABAB receptor agonists also exhibit a similar effect by increasing ERK(1/2) phosphorylation in brain tissue as a protective mechanism [33]. Activation of the MAPK subfamilies ERK (1/2), JNK, and p38 MAPK plays a crucial role in expressing inflammatory cytokines (TNF-α, IL-1β, and IL-6) in CFA-stimulated rats. The serum and DRG concentrations of IL-6, IL-10, and TNF-α exhibited a significant decrease after treatment with LED. Previous studies have also reported that the levels of inflammatory cytokines, including TNF-α, IL-1β, and IL-6, were changed when cells were stimulated externally[31,33].

Wnt/β-catenin, part of one of the classical pathways, includes the Wnt/β-Catenin pathway, the atypical Wnt/Planar cell polarity, and the Wnt/Calcium pathway. β-catenin, a downstream molecule of GSK3β, is a key protein in this pathway [34]. Non-phosphorylated GSK3β can induce the phosphorylation and degradation of β-catenin in the cytoplasm. Inhibition of GSK3β blocks the phosphorylation of β-catenin and prevents its degradation [35]. Wnt signaling pathways are aberrantly activated in the sciatic nerve, DRG, and SCDH in rodent models of chronic pain. Thus, Wnt signaling is a prospective therapeutic target for chronic pain [36]. LED and EA treatment greatly inhibited the CFA-induced increase in GK3β protein expression compared with that in the CFA group in the present study, while decreasing Wnt and β-catenin protein expression. However, the detailed mechanisms through which LED and EA regulate these pathways must be explored further. Taken together, our research indicates that activating the p38 MAPK and Wnt/β-catenin signaling pathway might contribute to the development of inflammatory pain by modulating the expression of proinflammatory cytokines, such as IL-6 and TNF-α. As we know, optogenetics has been used clinically. Several clinical trials on optogenetic therapy are ongoing, with one literature reported that patients with retinitis pigmentosa are treated with photogenetics [37]. However, clinical trials have not been reported regarding optogenetics treating inflammatory pain. Further clinical studies are warranted, and the best choice for specific opsins, and continued improvements in gene and light delivery systems should be explored.

## 5 Conclusion

**Fig. 8:**
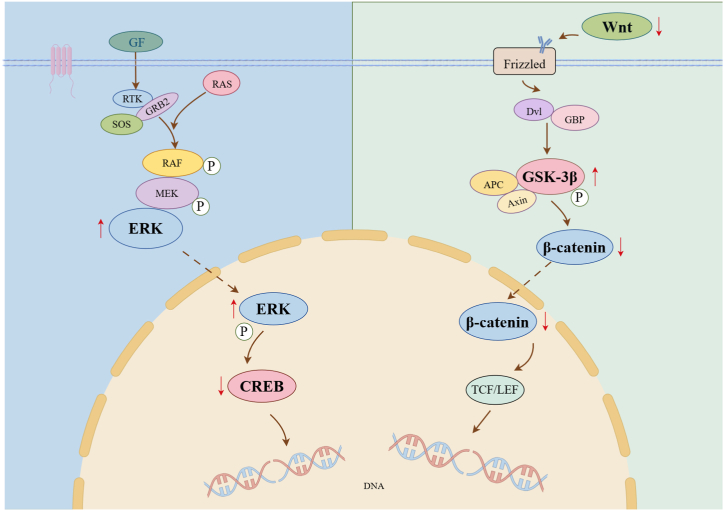
Outline of Optogenetic stimulation of the “Zusanli” acupoint alleviated the inflammatory pain through active Wnt/β-Catenin and MAPK signaling pathway in rats.

In conclusion, the present study demonstrated that optogenetic stimulation targeting GABA projections to the “Zusanli” acupoints effectively delivered rAAV9-GAD1-ChR2-mCherry to the SCDH and DRG, with a lesser extent to the toes, thereby eliciting an analgesic effect in CFA-treated rats. This effect was akin to that of acupuncture. Optogenetic manipulation at the “Zusanli” acupoints may alleviate inflammatory pain and inhibit inflammation by activating the p38 MAPK and Wnt signaling pathways. The present study's findings highlight the utility of pathway-selective optogenetics in revealing the neural mechanisms underlying the effects of acupuncture (Fig. 8).

## CRediT authorship contribution statement

**Rong Chen:** Writing – review & editing, Writing – original draft, Visualization, Validation, Supervision, Software, Data curation, Conceptualization. **Meng Li:** Writing – original draft, Visualization, Supervision, Funding acquisition, Formal analysis, Conceptualization. **Mingxing Ding:** Writing – review & editing, Data curation.

## Ethics approval and consent to participate

The Animals Ethics Committee of Huazhong Agriculture University approved this study (ID Number:HZAURA-2020-642) and these experiments were conducted according to established animal welfare guidelines.

## Funding

This study was supported by the National Science Foundation of China (grant numbers 31672615 and 31472246).

## Declaration of competing interest

The authors declare that they have no known competing financial interests or personal relationships that could have appeared to influence the work reported in this paper.
