# Supplementary material for: Optogenetic stimulation of the “Zusanli” acupoint alleviates inflammatory pain through active Wnt/β-Catenin and MAPK signaling pathway in rats
Source: Heliyon. 2024 Oct 30;10(21):e39992. doi: 10.1016/j.heliyon.2024.e39992 (PMC11566834; doi:10.1016/j.heliyon.2024.e39992)
Supplement: Multimedia component 1 [file mmc1.docx]

**Optogenetic stimulation of the “Zusanli” acupoint alleviates inflammatory pain through active Wnt/β-Catenin and MAPK signaling pathway in rats**

Rong Chen^1,2^, Meng Li^1^, Mingxing Ding^1^ *

^1^ College of Veterinary Medicine, Huazhong Agricultural University, Wuhan 430070, People’s Republic of China

^2^ College of Animal Science and Technology, Tarim University, Alar, Xinjiang 843300

*Corresponding author:

Mingxing Ding

College of Veterinary Medicine, Huazhong Agricultural University, Wuhan 430070, People’s Republic of China

Tel: 027-87286251

E-mail: [dmx@mail.hzau.edu.cn](mailto:dmx@mail.hzau.edu.cn)

**Contents**

**Figure 1C SDS-PAGE electrophoresis of the bands**

**Figure 5C Western blotting images of GAT1 in rats from different groups**

**Figure 6C Western blotting images of GAD65 in rats from different groups**

**Figure 7A Western blot analysis revealed that LED and EA reduce levels of ERK1/2, pERK1/2, CREB and pCREB protein in the SCDH 72 hours after CFA injection**

**
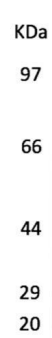

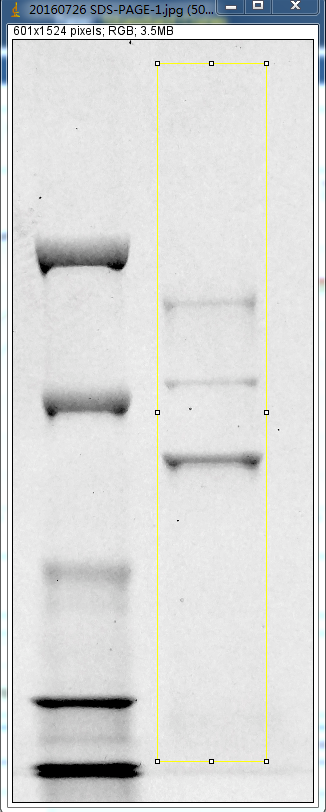

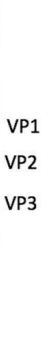
**

**Figure 1C : 10% SDS-PAGE electrophoresis of the bands**

**
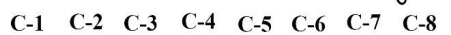
**

**
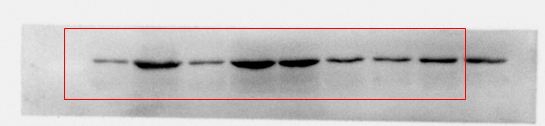
**

**Figure 5C DRG-GAT1**

**
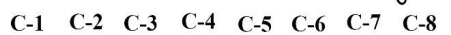
**

**
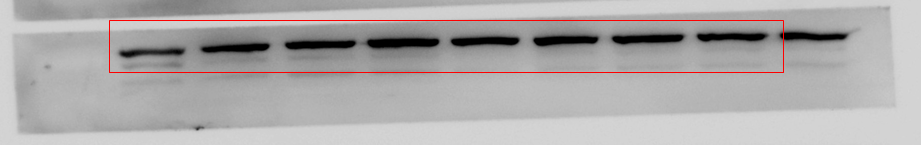
**

**Figure 5C DRG-GAPDH**

**
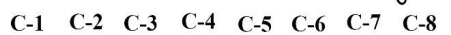
**

**
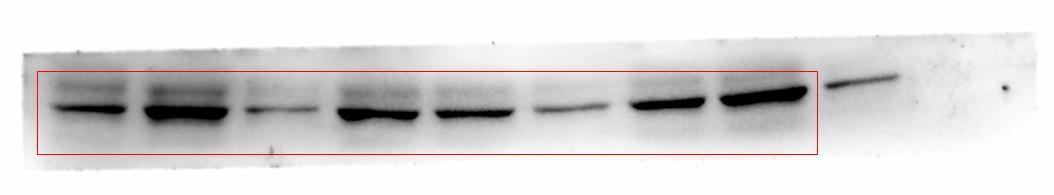
Figure 5C SCDH-GAT1**

**
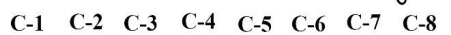
**

**
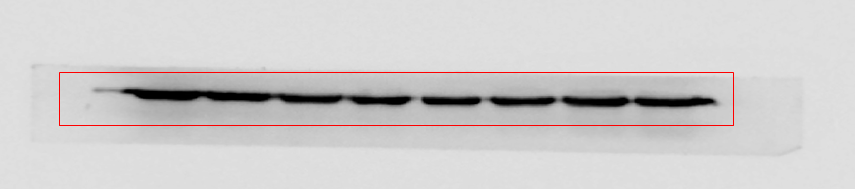
**

**Figure 5C SCDH-GAPDH**

**Figure 5C Western blotting images of GAT1 in rats from different groups**

C-1:Control(saline), C-2:CFA, C-3:CFA+EA, C-4:CFA+ChR2+LED (15 Hz)+ Dic, C-5:CFA+rAAV+LED (15 Hz), C-6:CFA+ChR2+LED (2 Hz), C-7:CFA+ChR2+LED (15 Hz), and C-8:CFA+ChR2+LED (60 Hz).

**
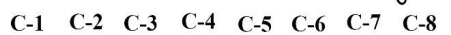
**


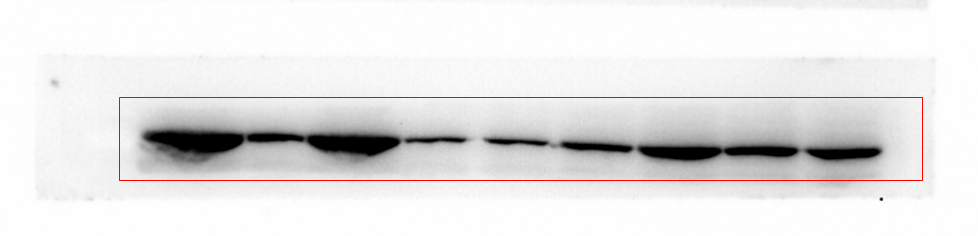


**Figure 6C DRG-GAD65**

**
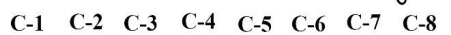
**

**
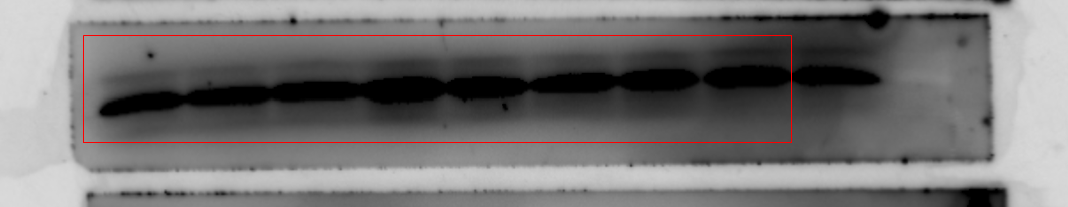
**

**Figure 6C DRG-GAPDH**

**
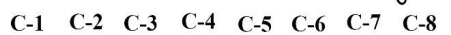
**

**
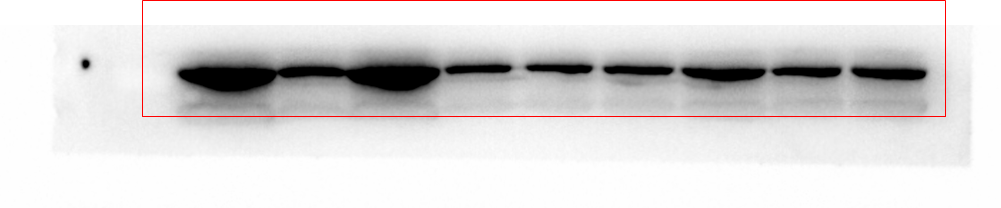
**

**Figure 6C SCDH-GAD65**

**
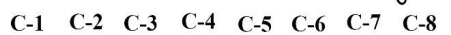
**

**
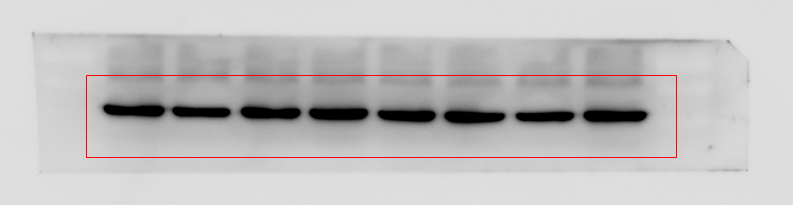
**

**Figure 6C SCDH-GAPDH**

**Figure 6C Western blotting images of GAD65 in rats from different groups**

C-1:Control(saline), C-2:CFA, C-3:CFA+EA, C-4:CFA+ChR2+LED (15

Hz)+Dicentrine (Dic), C-5:CFA+rAAV+LED (15 Hz), C-6:CFA+ChR2+LED (2 Hz), C-7:CFA+ChR2+LED (15 Hz), and C-8:CFA+ChR2+LED (60 Hz).

**
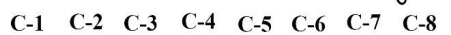
**

**
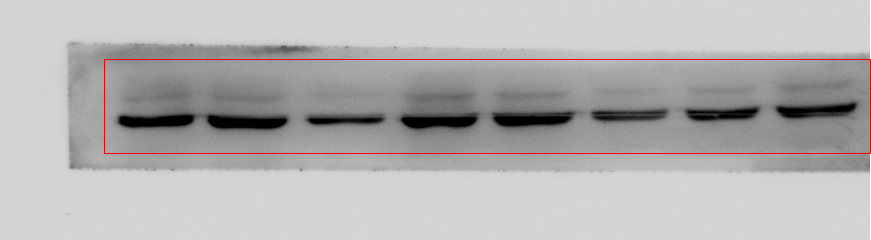
**

**Figure 7A CREB**

**
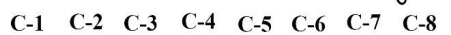
**

**
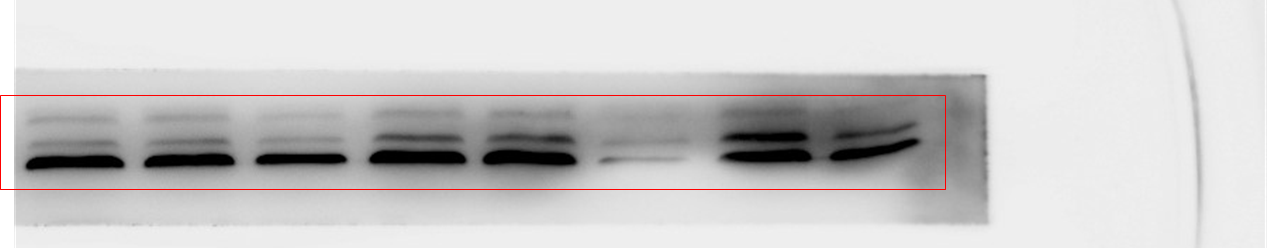
**

**Figure 7A ERK1/2**

**
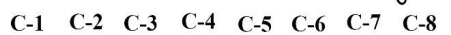
**

**
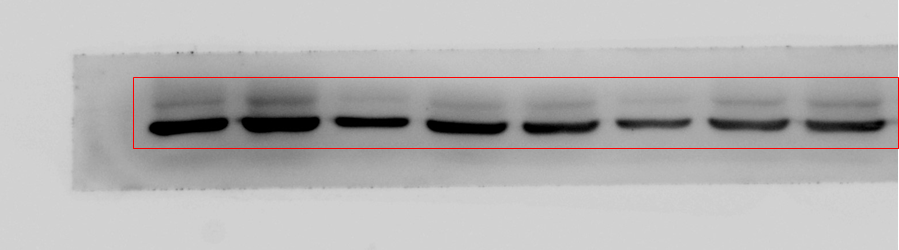
**

**Figure 7A P-CREB**

**
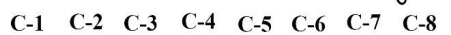
**

**
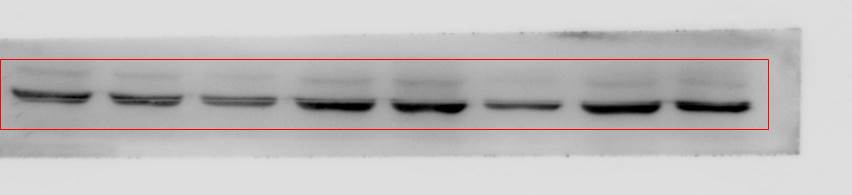
Figure 7A P-ERK**

**
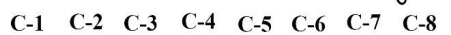
**

**
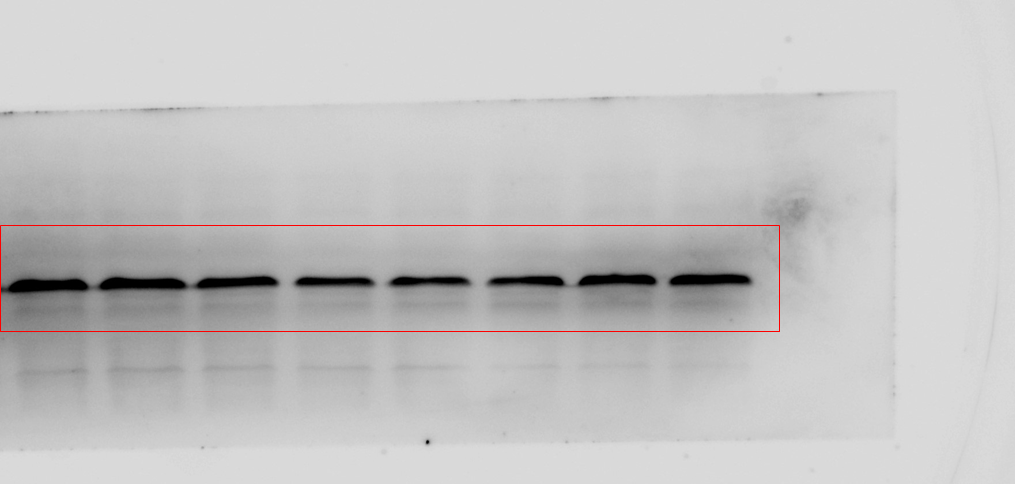
**

**Figure 7A β-actin**

**Figure 7A Western blot analysis revealed that LED and EA reduce levels of ERK1/2, pERK1/2, CREB and pCREB protein in the SCDH 72 hours after CFA injection**

C-1:Control(saline), C-2:CFA, C-3:CFA+EA, C-4:CFA+ChR2+LED (15

Hz)+Dicentrine (Dic), C-5:CFA+rAAV+LED (15 Hz), C-6:CFA+ChR2+LED (2 Hz), C-7:CFA+ChR2+LED (15 Hz), and C-8:CFA+ChR2+LED (60 Hz).
