# Supplementary material for: Optogenetic stimulation of the “Zusanli” acupoint alleviates inflammatory pain through active Wnt/β-Catenin and MAPK signaling pathway in rats
Source: Heliyon. 2024 Oct 30;10(21):e39992. doi: 10.1016/j.heliyon.2024.e39992 (PMC11566834; doi:10.1016/j.heliyon.2024.e39992)
Supplement: Multimedia component 2 [file mmc2.docx]

**Optogenetic stimulation of the “Zusanli” acupoint alleviates inflammatory pain through active Wnt/β-Catenin and MAPK signaling pathway in rats**

Rong Chen^1,2^, Meng Li^1^, Mingxing Ding^1^ *

^1^ College of Veterinary Medicine, Huazhong Agricultural University, Wuhan 430070, People’s Republic of China

^2^ College of Animal Science and Technology, Tarim University, Alar, Xinjiang 843300

*Corresponding author:

Mingxing Ding

College of Veterinary Medicine, Huazhong Agricultural University, Wuhan 430070, People’s Republic of China

Tel: 027-87286251

E-mail: [dmx@mail.hzau.edu.cn](mailto:dmx@mail.hzau.edu.cn)

**Contents**

Figure S1 Paw Edema of rats in each group at different time points

Figure S2 Western blotting test of GAT1 expression in DRG and SCDH of rats

Figure S3 Immunofluorescence images of LED and EA on rats' GAT1 expression in DRG and SCDH

Figure S4 qRT-PCR results of the effect of LED and EA on rats' GAT3 expression in DRG and SCDH

Figure S5 Western blotting results of the effect of LED and EA on GAT3 expression in DRG and SCDH

**Figure S6 Immunofluorescence of LED and EA on rats' GAT3 expression in DRG and SCDH.**

Figure S7 Western blotting results of the effect of LED and EA on GAD65 expression in DRG and SCDH

**Figure S8 Immunofluorescence images of LED and EA on rats' GAD65 expression in DRG and SCDH.**

**Figure S9 qRT-PCR results of the effect of LED and EA on rats' *GAD67* expression in DRG and SCDH.**

**Figure S10 Western blotting results of the effect of LED and EA on GAD67 expression in DRG and SCDH**

**Figure S11 Immunofluorescence of LED and EA on rats' GAD67 expression in DRG and SCDH**

**Figure S12 Effect of LED and EA treatment on TNF-α, IL-6 and IL-β in the DRG and serum of CFA-injected rats**

Figure S13 Effect of LED and EA treatment on Wnt/β-catenin signaling pathway in the SCDH of CFA-injected rats


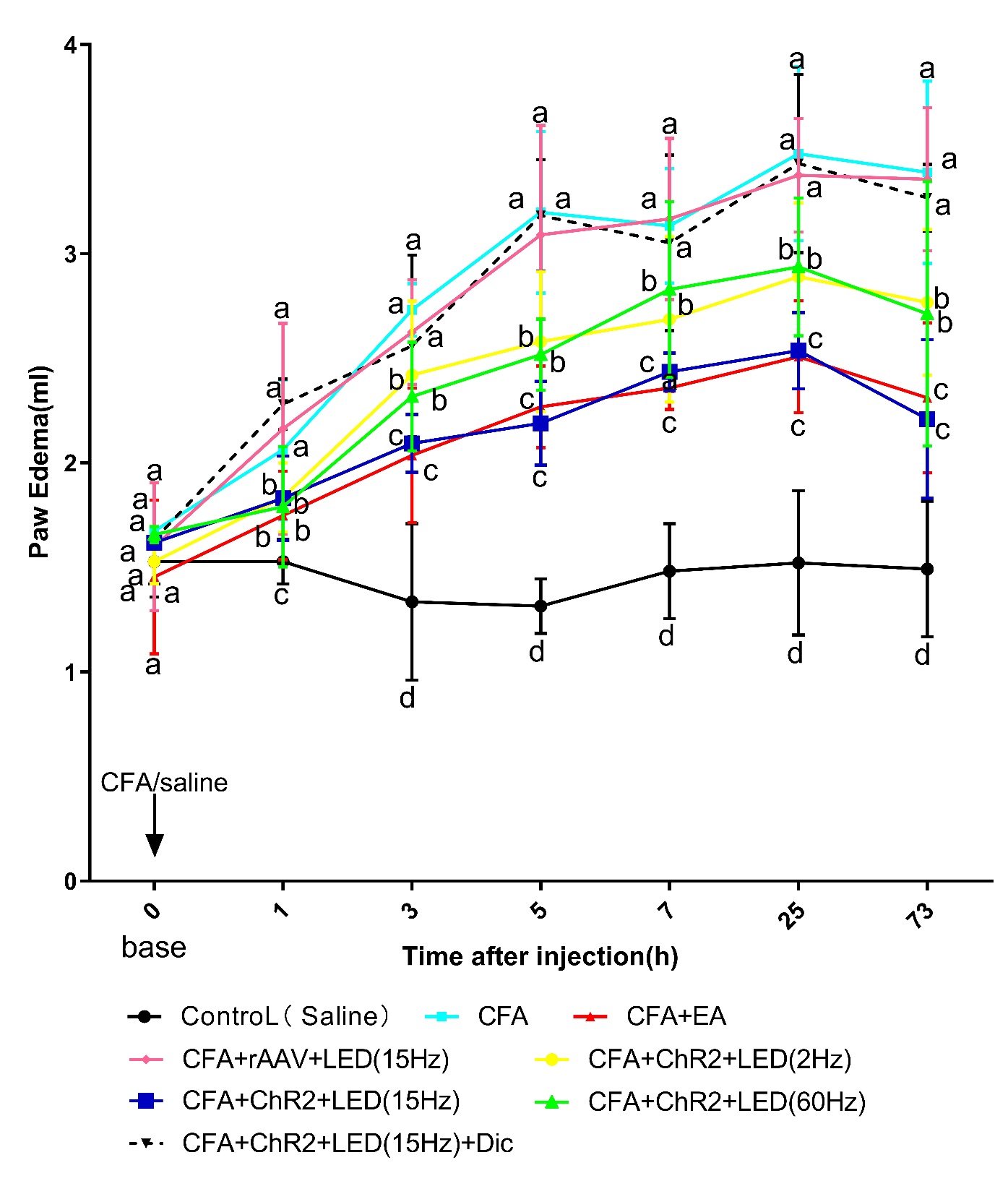


**Figure S1 Paw Edema of rats in each group at different time points.**

**Paw Edema** value of rats in each group at different time-points. Different letters were significantly different in each group at different time points (*P* < 0.05).


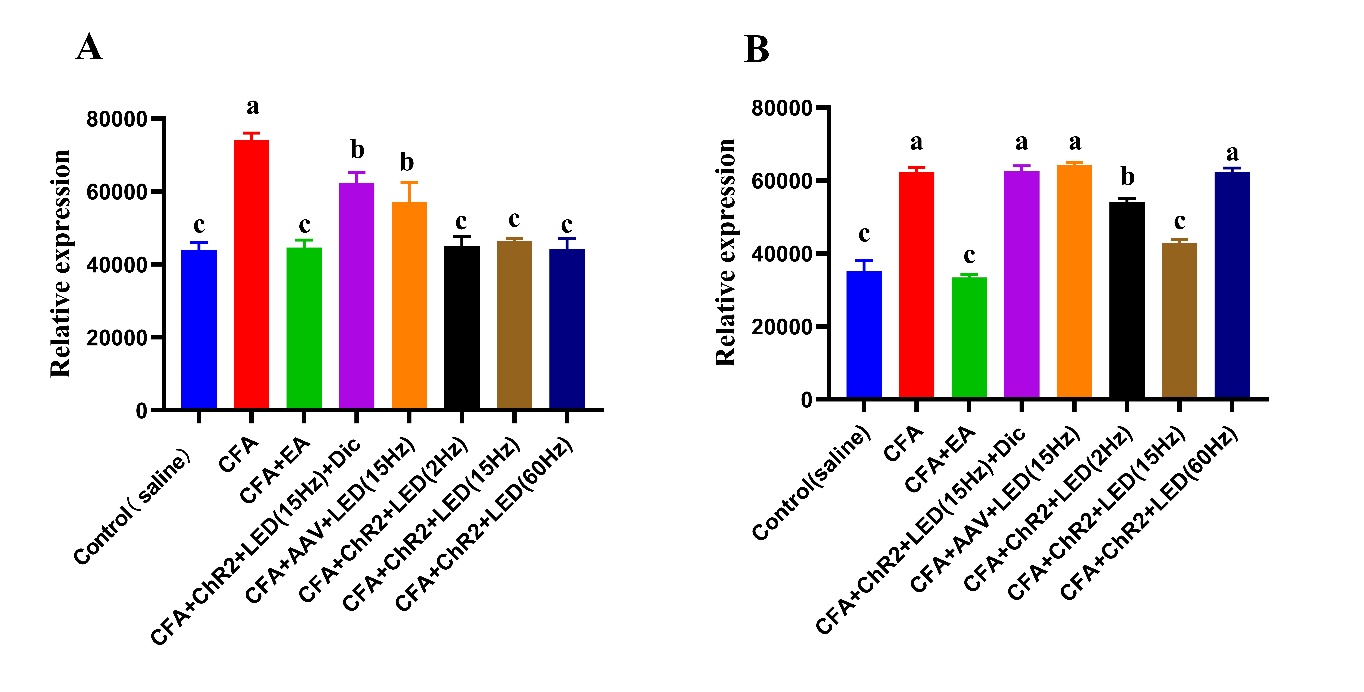


**Figure S2 Western blotting test of GAT1 expression in DRG and SCDH of rats.**

A. Western blotting results of GAT1 expression in the DRG of rats; B. Western blotting results of GAT1 expression in the SCDH of rats; Different letters were significantly different in each group (*P* < 0.05).


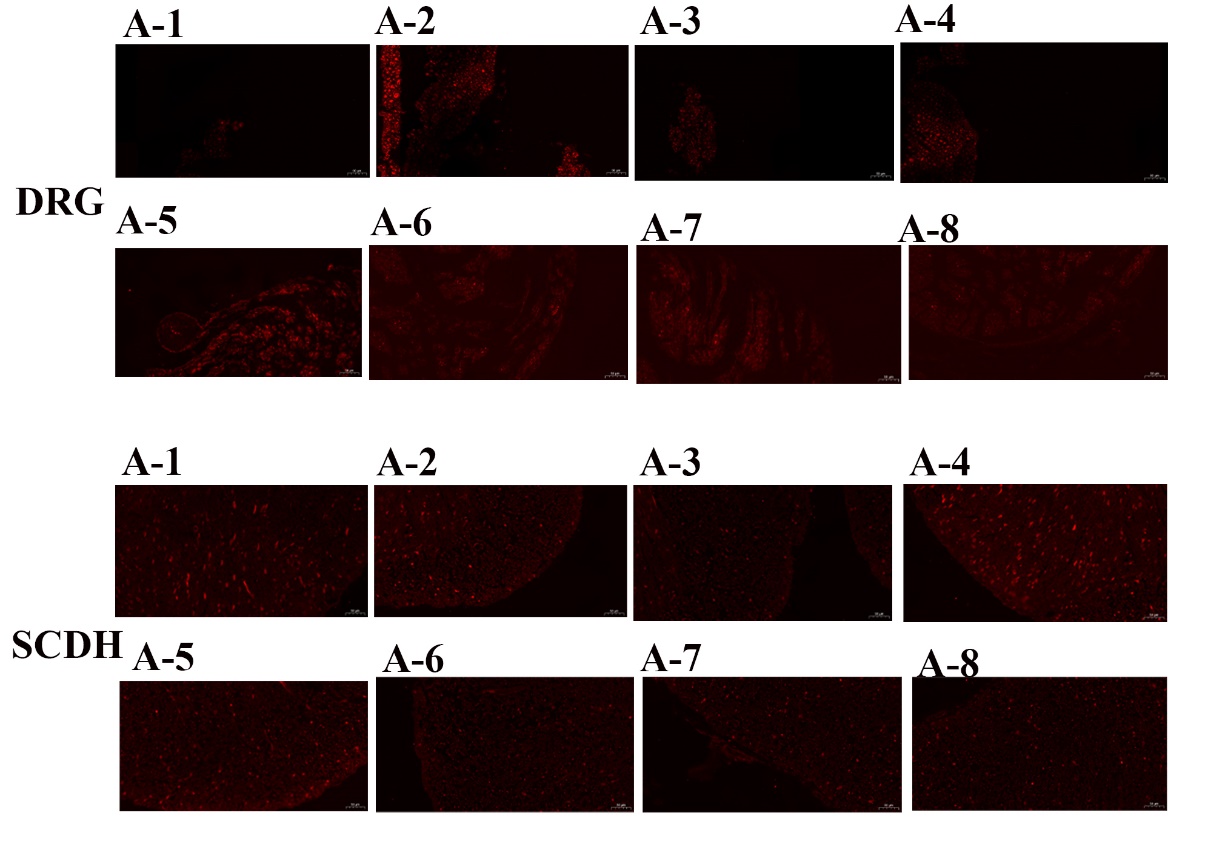


**Figure S3 Immunofluorescence images of LED and EA on rats' GAT1 expression in DRG and SCDH.**

A-1:Control(saline), A-2:CFA, A-3:CFA+EA, A-4:CFA+ChR_2_+LED (15 Hz)+ Dic, A-5:CFA+rAAV+LED (15 Hz), A-6:CFA+ChR_2_+LED (2 Hz), A-7:CFA+ChR_2_+LED (15 Hz), and A-8:CFA+ChR_2_+LED (60 Hz)


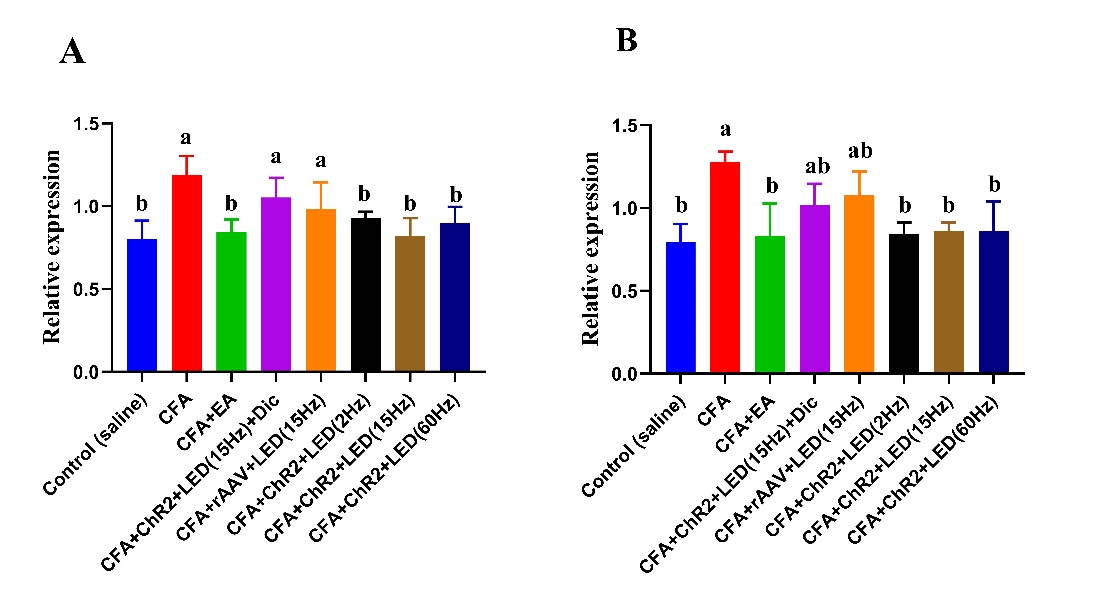


**Figure S4 qRT-PCR results of the effect of LED and EA on rats' GAT3 expression in DRG and SCDH.**

A. qRT-PCR results of *GAT3* expression in the DRG of rats; B. qRT-PCR results of *GAT3* expression in the SCDH of rats; Different letters were significantly different in each group (*P* < 0.05).

**
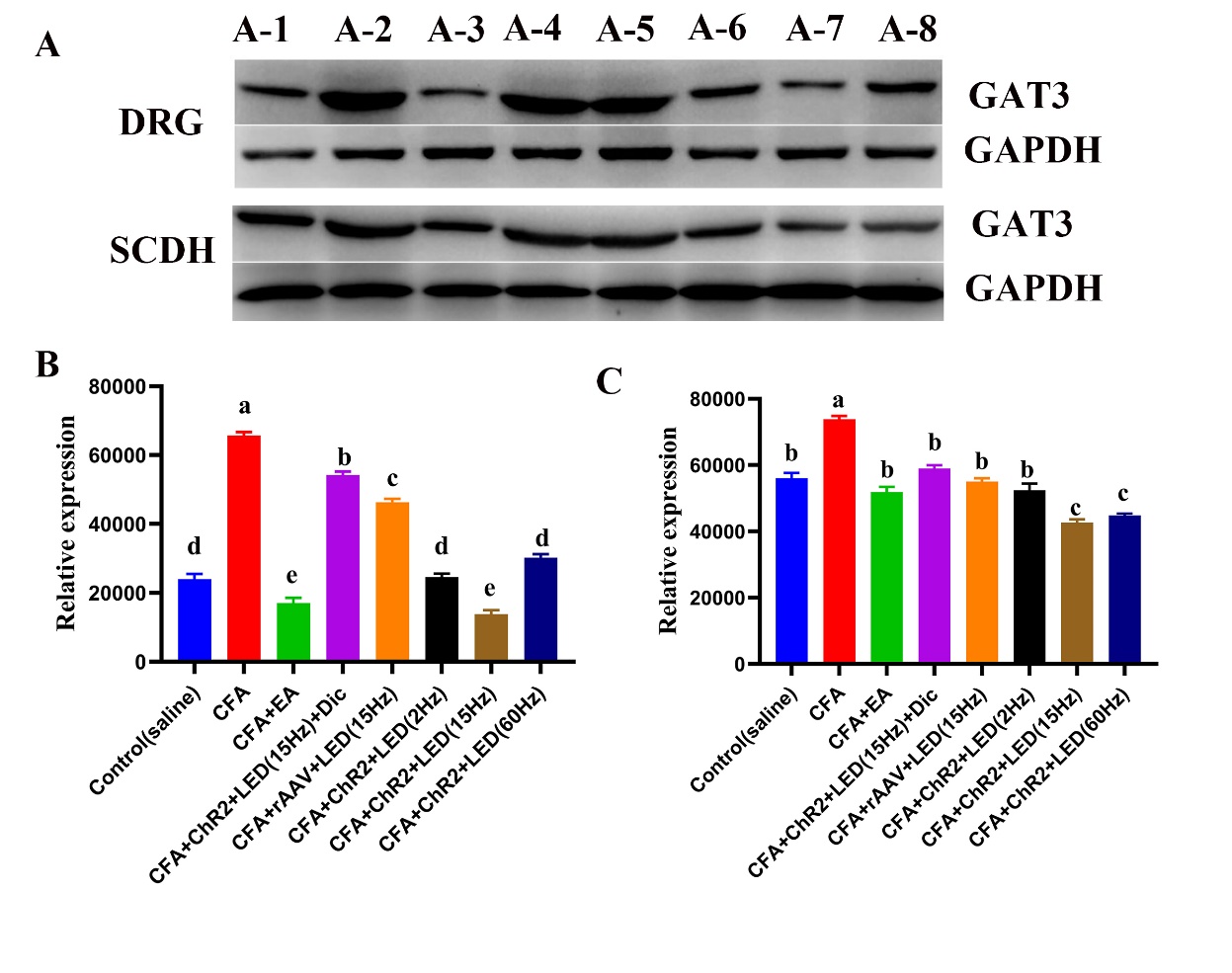
**

**Figure S5 Western blotting results of the effect of LED and EA on GAT3 expression in DRG and SCDH**

A. Western blotting images of GAT3 in rats from different groups; A-1: Control(saline), A-2:CFA, A-3:CFA+EA, A-4:CFA+ChR_2_+LED (15 Hz)+ Dic, A-5:CFA+rAAV+LED (15 Hz), A-6:CFA+ChR_2_+LED (2 Hz), A-7:CFA+ChR_2_+LED (15 Hz), and A-8:CFA+ChR_2_+LED (60 Hz); B. Western blotting results of *GAT3* expression in the DRG of rats; C. Western blotting qRT-PCR results of *GAT3* expression in the SCDH of rats. Different letters were significantly different in each group (*P* < 0.05).


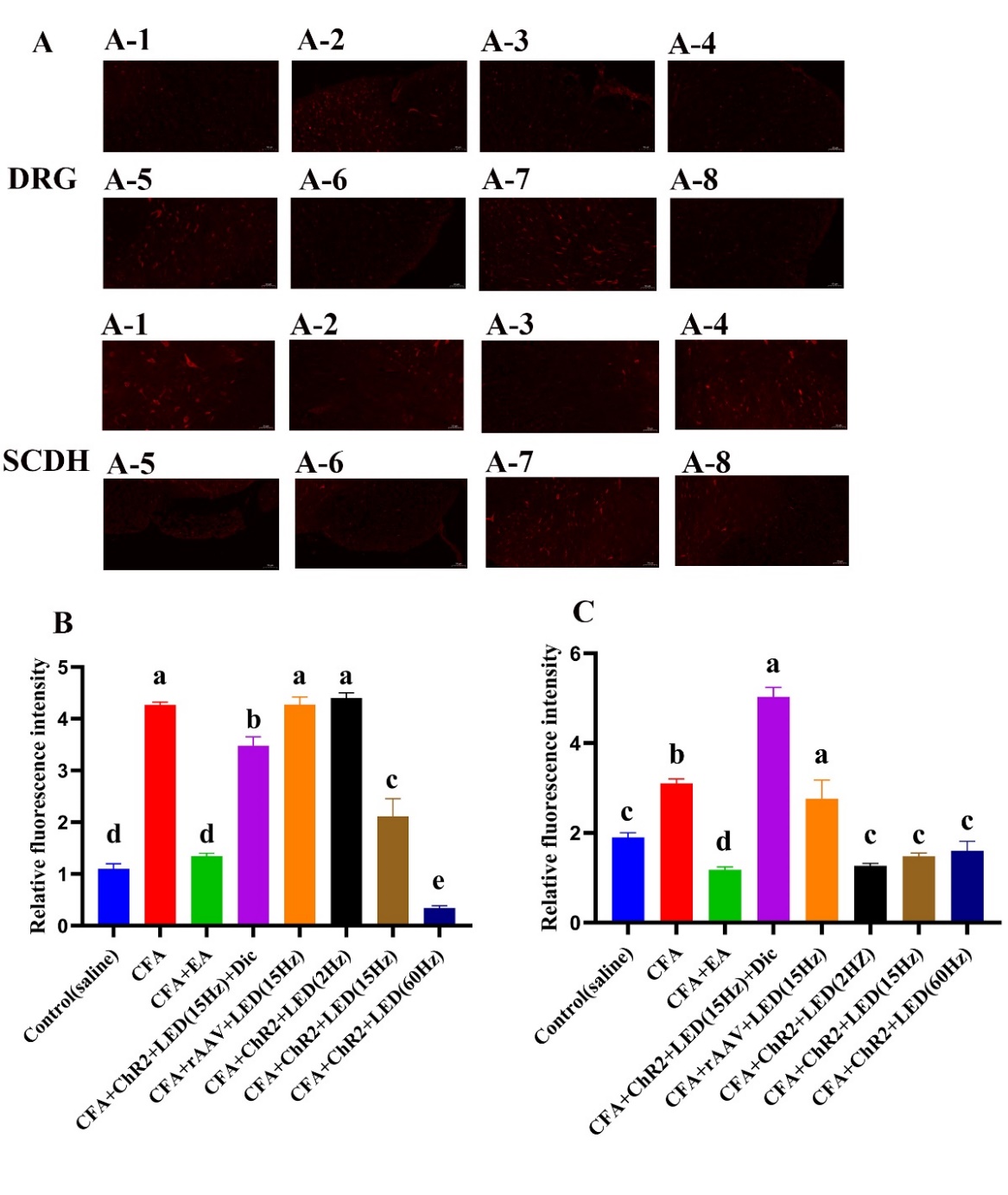


**Figure S6 Immunofluorescence of LED and EA on rats' GAT3 expression in DRG and SCDH.**

A. Immunofluorescence image of LED and EA on rats' GAT3 expression in DRG and SCDH. A-1:Control(saline), A-2:CFA, A-3:CFA+EA, A-4:CFA+ChR_2_+LED (15 Hz)+ Dic, A-5:CFA+rAAV+LED (15 Hz), A-6:CFA+ChR_2_+LED (2 Hz), A-7:CFA+ChR_2_+LED (15 Hz), and A-8:CFA+ChR_2_+LED (60 Hz) ; B. Fluorescence intensity of GAT3 expression in the DRG of rats; C. Fluorescence intensity of GAT3 expression in the SCDH of rats; Different letters were significantly different in each group (*P* < 0.05).


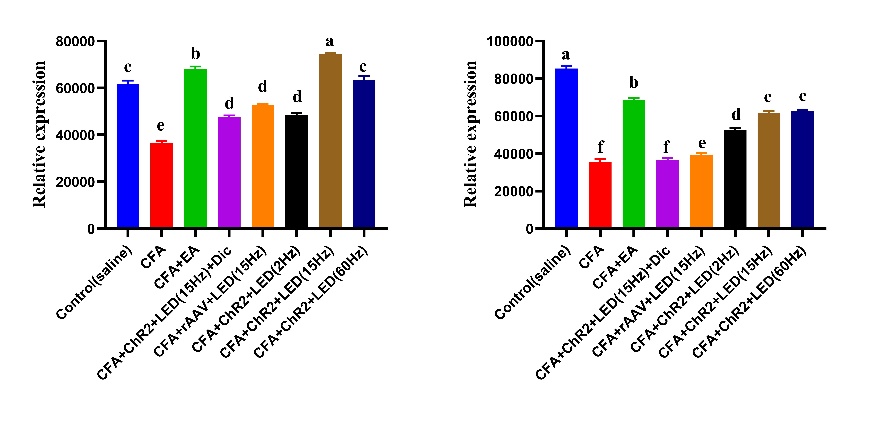


Figure S7 Western blotting results of the effect of LED and EA on GAD65 expression in DRG and SCDH.

A. Western blotting results of GAD65 expression in the DRG of rats; D. Western blotting results of GAD65 expression in the SCDH of rats. Different letters were significantly different in each group (*P* < 0.05).


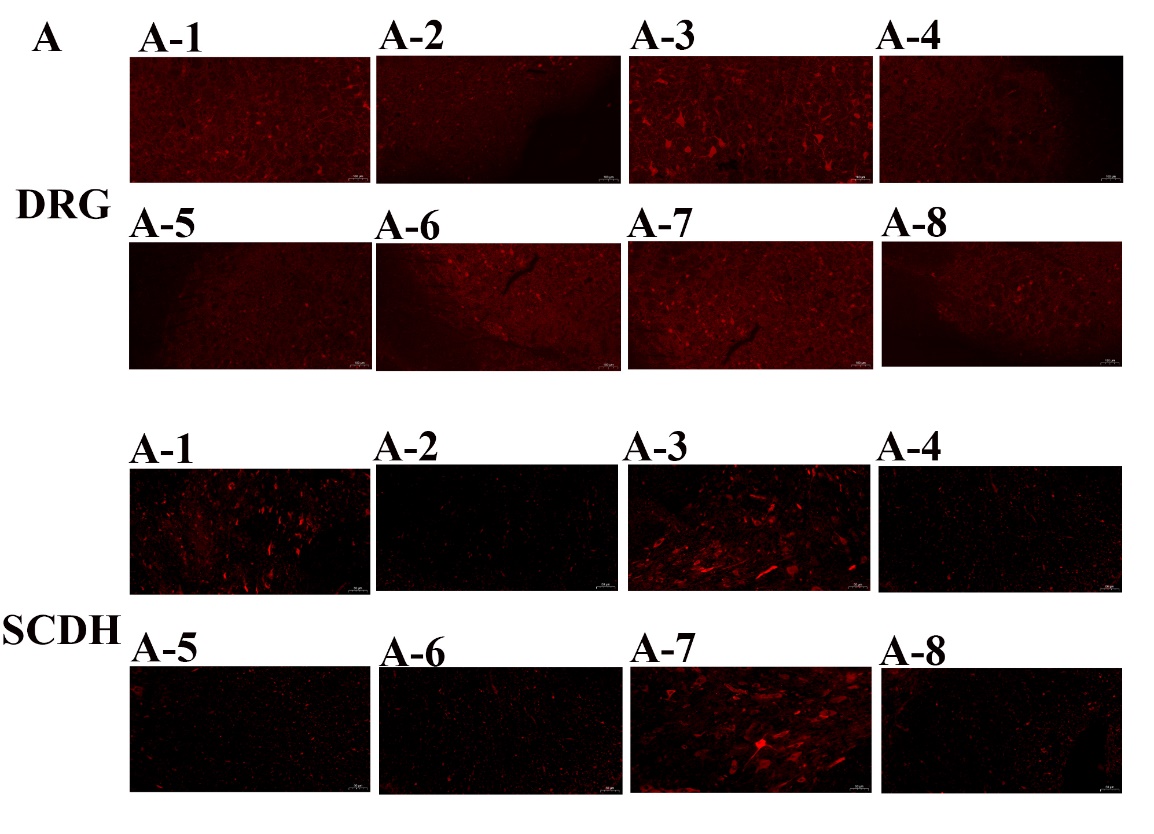


**Figure S8 Immunofluorescence images of LED and EA on rats' GAD65 expression in DRG and SCDH.**

A-1:Control(saline), A-2:CFA, A-3:CFA+EA, A-4:CFA+ChR_2_+LED (15 Hz)+ Dic, A-5:CFA+rAAV+LED (15 Hz), A-6:CFA+ChR_2_+LED (2 Hz), A-7:CFA+ChR_2_+LED (15 Hz), and A-8:CFA+ChR_2_+LED (60 Hz)


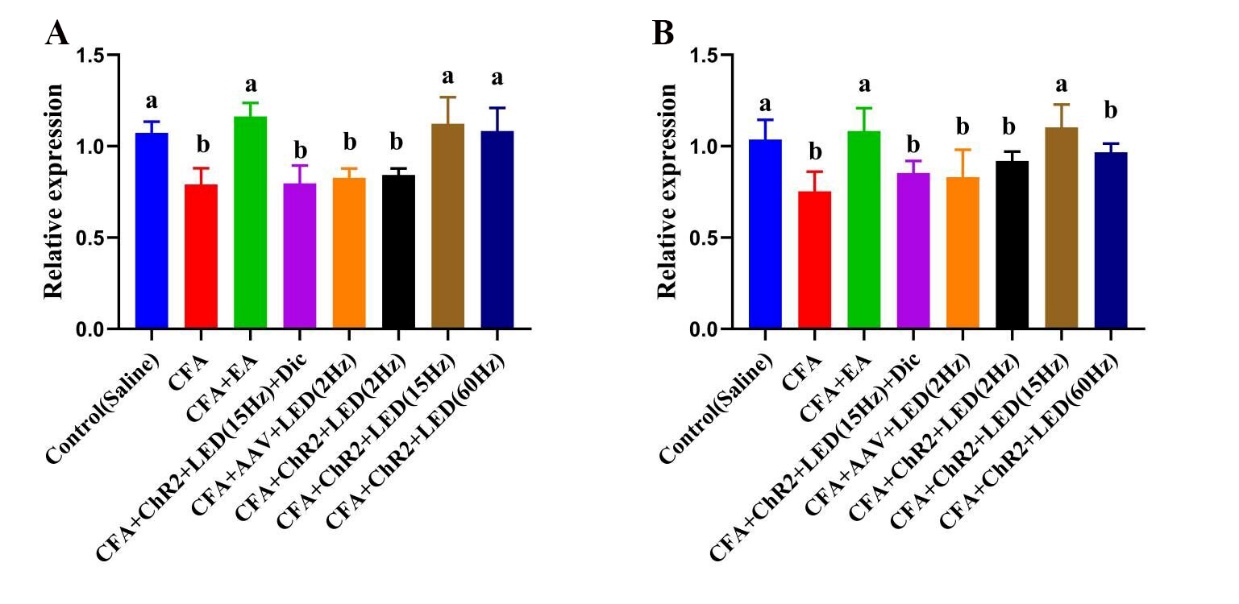


**Figure S9 qRT-PCR results of the effect of LED and EA on rats' *GAD67* expression in DRG and SCDH.**

A. qRT-PCR results of *GAD67* expression in the DRG of rats; B. qRT-PCR results of *GAD67* expression in the SCDH of rats; Different letters were significantly different in each group (*P* < 0.05).


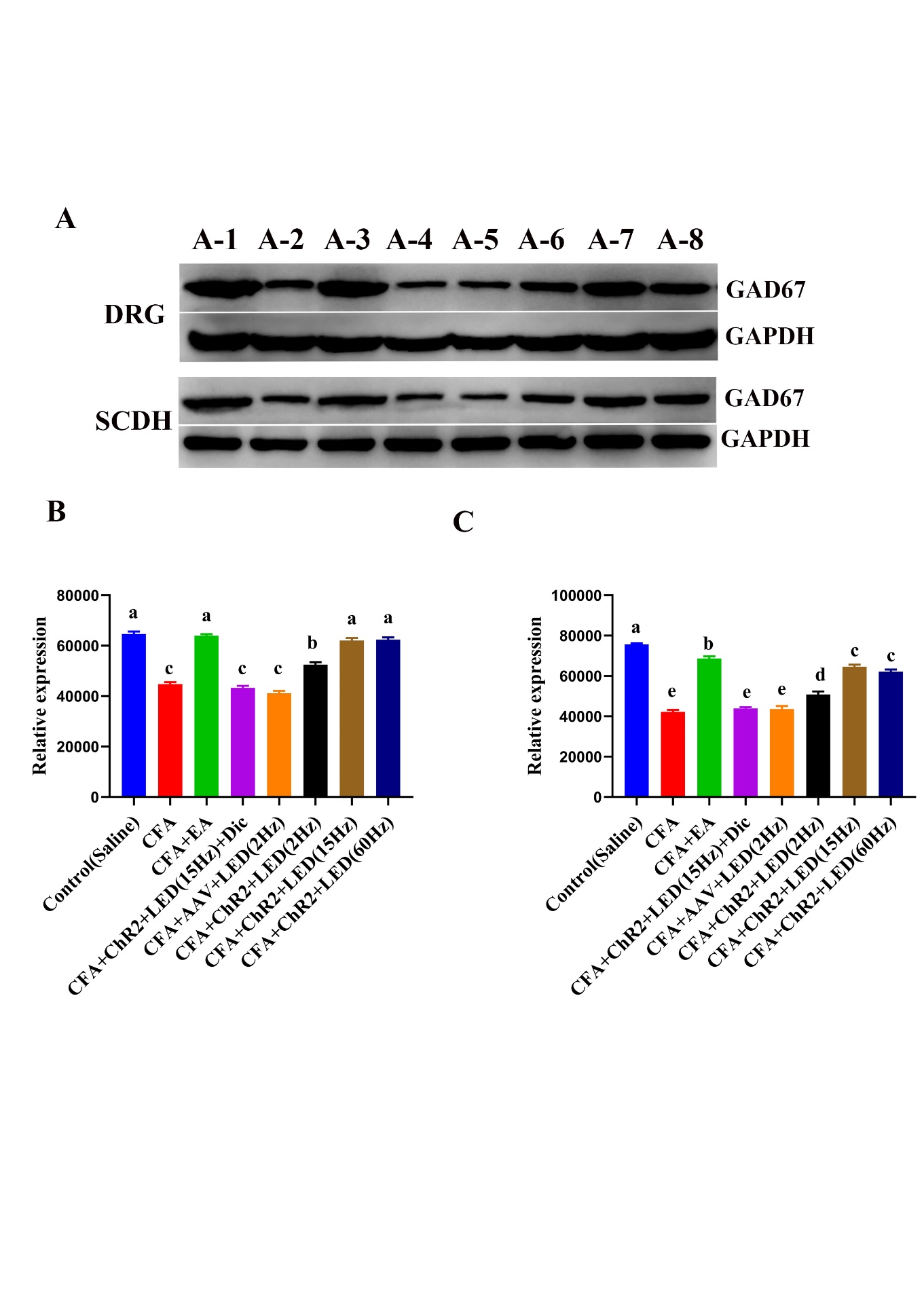


**Figure S10 Western blotting results of the effect of LED and EA on GAD67 expression in DRG and SCDH**

A. Western blotting images of GAD67 in rats from different groups. A-1:Control(saline), A-2:CFA, A-3:CFA+EA, A-4:CFA+ChR_2_+LED (15 Hz)+ Dic, A-5:CFA+rAAV+LED (15 Hz), A-6:CFA+ChR_2_+LED (2 Hz), A-7:CFA+ChR_2_+LED (15 Hz), and A-8:CFA+ChR_2_+LED (60 Hz); B. Western blotting results of GAD67 expression in the DRG of rats; C. Western blotting qRT-PCR results of GAD67 expression in the SCDH of rats

**
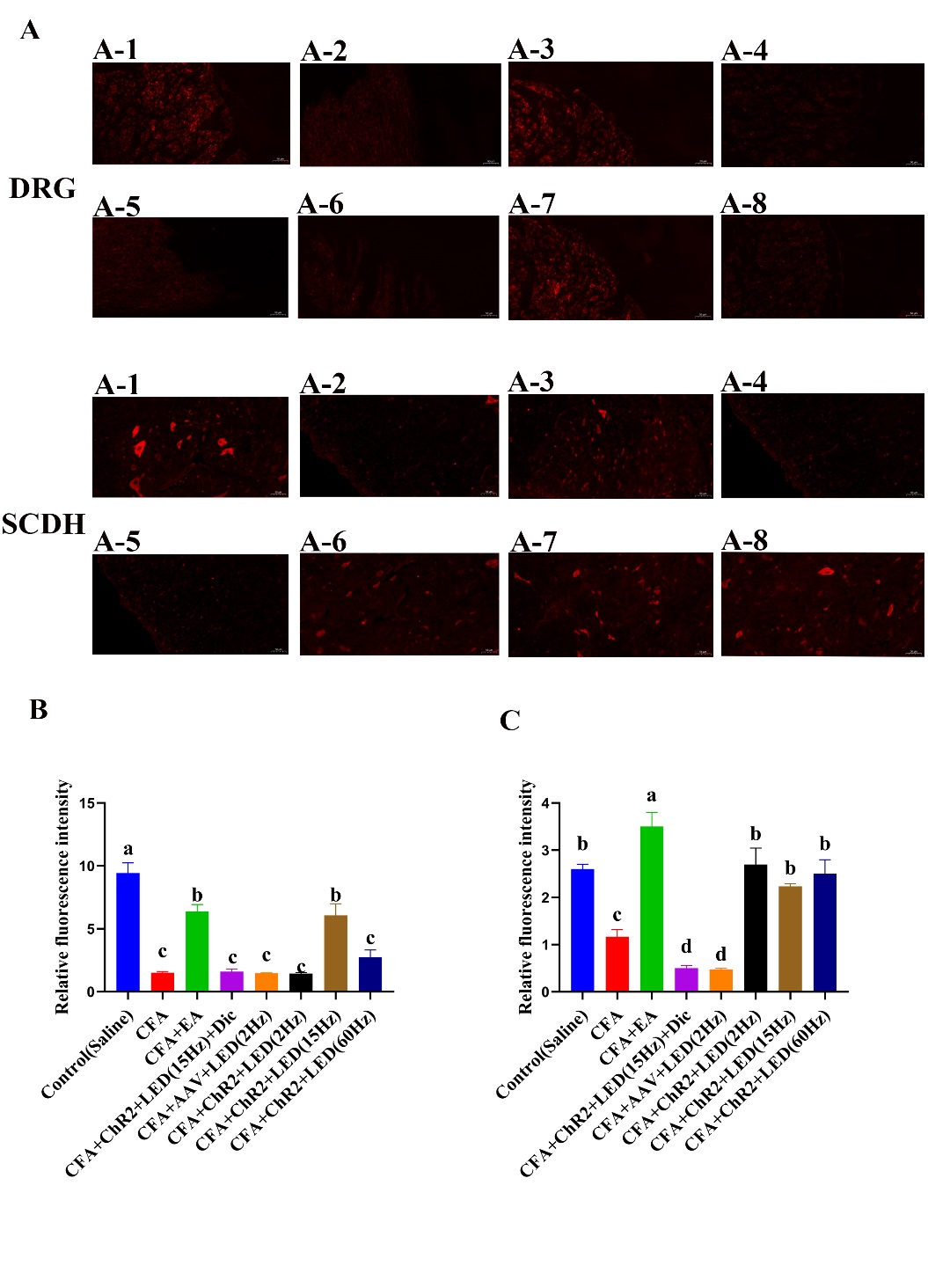
**

**Figure S11 Immunofluorescence of LED and EA on rats' GAD67 expression in DRG and SCDH.**

A: Immunofluorescence image of LED and EA on rats' GAD67 expression in DRG and SCDH.

A-1:Control(saline), A-2:CFA, A-3:CFA+EA, A-4:CFA+ChR_2_+LED (15 Hz)+Dicentrine (Dic), A-5:CFA+rAAV+LED (15 Hz), A-6:CFA+ChR_2_+LED (2 Hz), A-7:CFA+ChR_2_+LED (15 Hz), and A-8:CFA+ChR_2_+LED (60 Hz); B. Fluorescence intensity of GAD67 expression in the DRG of rats; C. Fluorescence intensity of GAD67 expression in the SCDH of rats; Different letters were significantly different in each group (*P* < 0.05).

**
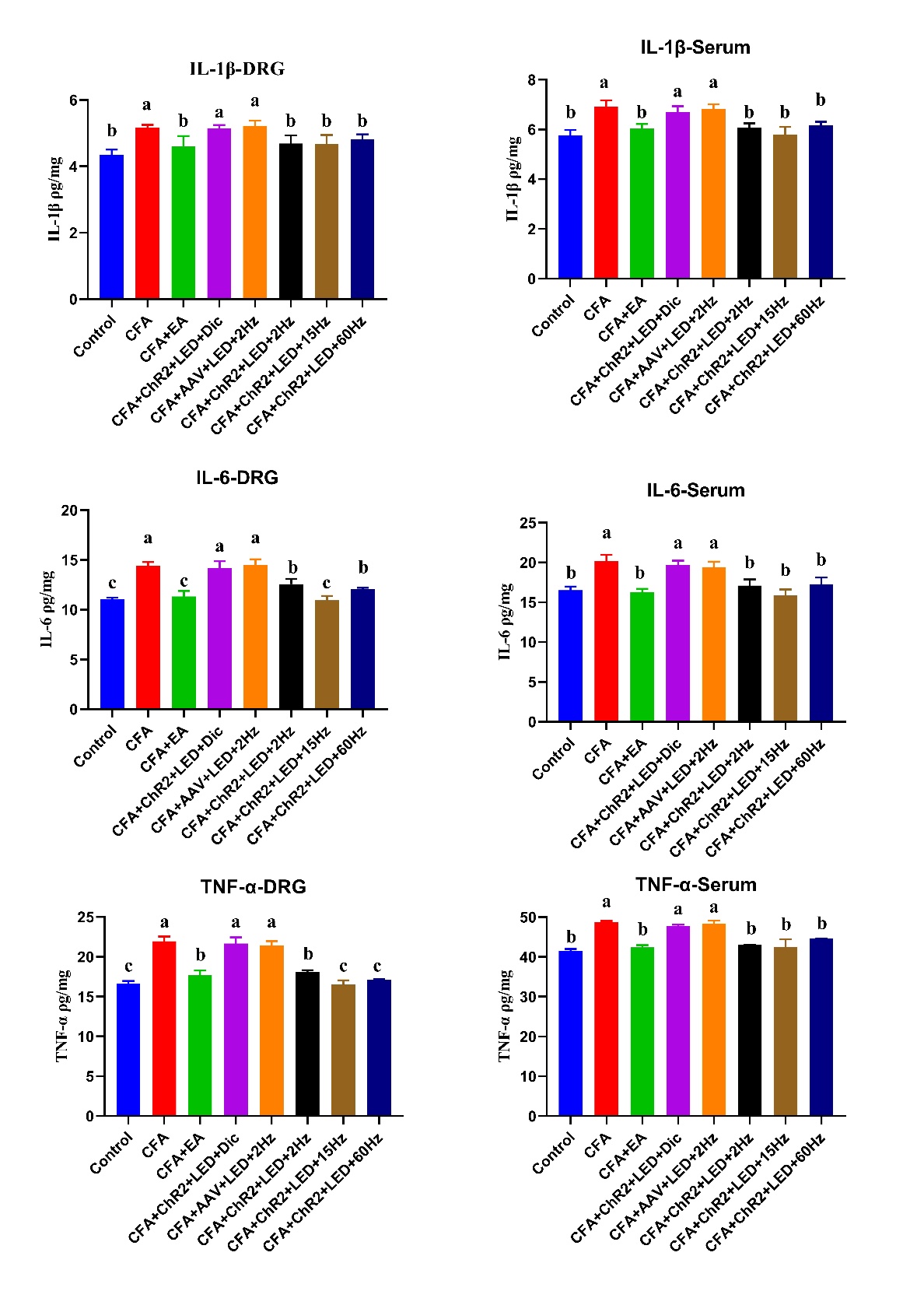
**

**Figure S12 Effect of LED and EA treatment on TNF-α, IL-6 and IL-β in the DRG and serum of CFA-injected rats.**

Each group's letters differed significantly at different time points (*P < 0.05*).


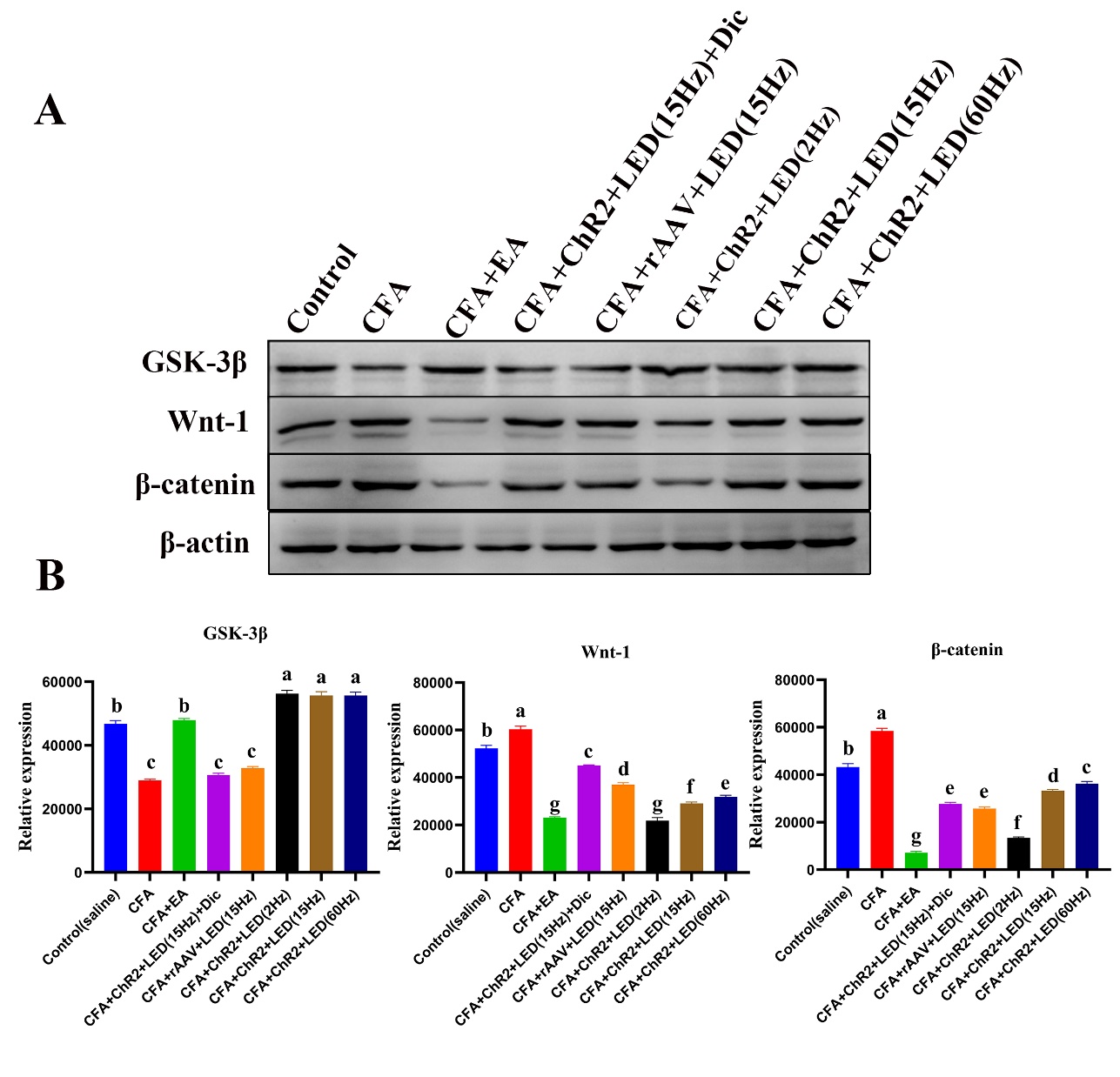


**Figure S13 Effect of LED and EA treatment on Wnt/β-catenin signaling pathway in the SCDH of CFA-injected rats.**

A. Western blot analysis revealed that LED and EA reduce levels of GK3β, Wnt, CREB and β-catenin protein in the SCDH 72 hours after CFA injection. B. Quantification of GK3β, pCREB and Wnt protein normalized against β-actin. Each bar represents mean ± SEM (n = 3). Bars with different letters differ significantly (*P*＜0.05)
